# Steps of actin filament branch formation by Arp2/3 complex investigated with coarse-grained molecular dynamics

**DOI:** 10.3389/fcell.2023.1071977

**Published:** 2023-01-17

**Authors:** Shuting Zhang, Dimitrios Vavylonis

**Affiliations:** ^1^ Department of Physics, Lehigh University, Bethlehem, PA, United States; ^2^ Center for Computational Biology, Flatiron Institute, New York, NY, United States

**Keywords:** actin cytoskeleton, ARP2/3 complex, molecular dynamics, coarse-grained modeling, branch nucleation

## Abstract

The nucleation of actin filament branches by the Arp2/3 complex involves activation through nucleation promotion factors (NPFs), recruitment of actin monomers, and binding of the complex to the side of actin filaments. Because of the large system size and processes that involve flexible regions and diffuse components, simulations of branch formation using all-atom molecular dynamics are challenging. We applied a coarse-grained model that retains amino-acid level information and allows molecular dynamics simulations in implicit solvent, with globular domains represented as rigid bodies and flexible regions allowed to fluctuate. We used recent electron microscopy structures of the inactive Arp2/3 complex bound to NPF domains and to mother actin filament for the activated Arp2/3 complex. We studied interactions of Arp2/3 complex with the activating VCA domain of the NPF Wiskott-Aldrich syndrome protein, actin monomers, and actin filament. We found stable configurations with one or two actin monomers bound along the branch filament direction and with CA domain of VCA associated to the strong and weak binding sites of the Arp2/3 complex, supporting prior structural studies and validating our approach. We reproduced delivery of actin monomers and CA to the Arp2/3 complex under different conditions, providing insight into mechanisms proposed in previous studies. Simulations of active Arp2/3 complex bound to a mother actin filament indicate the contribution of each subunit to the binding. Addition of the C-terminal tail of Arp2/3 complex subunit ArpC2, which is missing in the cryo-EM structure, increased binding affinity, indicating a possible stabilizing role of this tail.

## Introduction

The seven-subunit Arp2/3 complex is a main regulator of the actin cytoskeleton, playing important roles in cell motion, division, and many intracellular functions (Pollard, 2007; Gautreau et al., 2022; Lappalainen et al., 2022; Martin and Suzanne, 2022). Its Arp2 and Arp3 subunits can come together to provide a barbed end interface for the elongation of a new actin filament. The Arp2/3 complex can also bind to preexisting actin filaments (“mother filaments”) to nucleate the growth of new actin filament (“daughter filament”) at a characteristic 70° angle with respect to the mother. The Arp2/3 complex is thus a molecular machinery that provides the directionality, orientation, and large-scale architecture to the actin cytoskeleton that is necessary for its function. For example, in the lamellipodia at the leading edge of motile cells, Arp2/3 branching, in coordination with barbed end capping, leads to dendritic networks that self-organize into orientation patterns optimized for force generation against the opposing plasma membrane (Holz and Vavylonis, 2018).

Precise temporal and spatial control of Arp2/3-complex-mediated actin polymerization, of great importance to cells, is a complex process, many aspects of which are still actively researched. Classic biochemical studies (Pollard, 2007) showed that side branch nucleation of the Arp2/3 complex is catalyzed by the combined action of 1) nucleation promotion factors (NPFs) such as VCA containing V (also called WH2), C, and A domains, 2) free actin monomers, and 3) mother actin filaments. Additional Arp2/3 activation pathways, which don’t involve a mother actin filament, have also been described, for example through the WISH/DIP/SPIN90 proteins (Gautreau et al., 2022).

Several experimental studies showed that the Arp2/3 complex has two binding sites for the CA region of VCA (Padrick et al., 2008; Padrick et al., 2011; Ti et al., 2011; Boczkowska et al., 2014; Luan et al., 2018). Two locations of CA binding on the inactive Arp2/3 complex have been recently determined by cryo-EM studies (Zimmet et al., 2020). Considering that actin monomers bind to VCA’s V region, this study supported a detailed mechanistic activation pathway where a complex of Arp2/3 with two bound VCA carrying two actin monomers (as would occur near the plasma membrane with bound WASP containing VCA) is established prior to its binding to a mother actin filament and subsequent side branch elongation (Zimmet et al., 2020).

Another important recent advance in understanding the Arp2/3 complex activation mechanism has been the use of electron microscopy to characterize the structure of the activated complex, by cryo-ET of NIH-3T3 fibroblast cells (Fäßler et al., 2020), cryo-EM of activated Arp2/3 complex from *Bos taurus in vitro* (Ding et al., 2022), as well as yeast Arp2/3 bound to Dip1 (Shaaban et al., 2020). These studies showed the twisting conformational change of the complex that helps bring the Arp2 and Arp3 subunits closer together to stabilize the pointed end of the daughter filament.

Prior all-atom molecular dynamics (MD) simulations, without the benefit of the recent cryo-EM structures, have explored various aspects of Arp2/3 complex activation (Dalhaimer and Pollard, 2010; Goley et al., 2010; Pfaendtner et al., 2012; Laporte and Magistrato, 2021). However, the use of all atom computer simulations to help quantify the biophysical basis of Arp2/3 activation is limited by the large size of the Arp2/3 complex and of the associated mother actin filament. Its activation also includes multiple binding events (of VCA, actin monomers, and mother filament), conformational changes, and flexible regions such as the VCA domain and the D-loop of actin. Motivated by recent coarse-grained (CG) MD studies of pure actin and formin-actin polymerization (Horan et al., 2018; Horan et al., 2020; Smith et al., 2021), here we explore the use of these methods to study Arp2/3 complex activation. We used the model A of Kim and Hummer (2008) (KH-A), which has much reduced computational cost compared to all atom simulations but still retains aminoacid specificity.

Considering the above recent experimental structural advances, as well as the most recently updated model of Arp2/3 complex activation pathway proposed by Zimmet et al. (2020), we performed simulations towards three main goals. First, we tested the extent to which the KH-A CG model is able to reproduce experimentally observed binding interactions among VCA, actin and Arp2/3 complex. Second, based on the success of the first goal, we used the CG model to explore complexes and interactions among components that have been proposed to exist as part of the activation pathway, but which haven’t yet been directly observed. In this way, we attempted to verify or challenge the activation mechanism proposed by Zimmet et al. (2020). Third, to provide suggestions for experimental tests by quantifying the strength of binding interactions that may reveal key components or pathways involved in Arp2/3 complex activation.

## Material and methods

### Coarse-grained molecular dynamics

We apply the Kim and Hummer (2008) Model A (KH-A), where each residue is represented by its C_*α*_ atom, as described in Horan et al. (2020); Smith et al. (2021). In the KH-A model, pairs of non-bonded residues interact through a Lennard-Jones (LJ)-type potential, either attractive or repulsive, based on the Miyazawa and Jernigan (1996) pairwise interaction matrix. There is no explicit solvent in the simulation systems. Debye-Hückel elactrostatics is used with uniform dielectric constant 80 and screening length 10 Å corresponding to ∼ 100 mM monovalent salt concentration. All LJ-like pairwise interactions were cutoff at a distance of three *σ*, where *σ* is the corresponding LJ parameter, while electrostatic interactions were cutoff at 35 Å.

During the simulations, protein domains with confirmed experimental structures are set as rigid and thus the interactions within these domains are neglected. The simulations didn’t include the nucleotides and associated divalent ion that are bound to Arp2, Arp3 or actin. Other flexible residues are connected to the neighboring residues by harmonic springs with equilibrium length 3.81 Å and spring constant 189 kcal/(mol Å^2^).

Simulations were performed using LAMMPS (Plimpton, 1995; in ’t Veld et al., 2008) and the KH-A model implementation of Smith et al. (2021) available at https://github.com/aah217/KH_LAMMPS. Depending on the system studied, serial simulations or replica exchange MD (REMD) simulations are performed. We used a time steps 10 fs. Our neighbor lists were updated every 1,000 steps. Langevin dynamics is applied with a damping parameter of 1,000 fs

**FIGURE 1:**
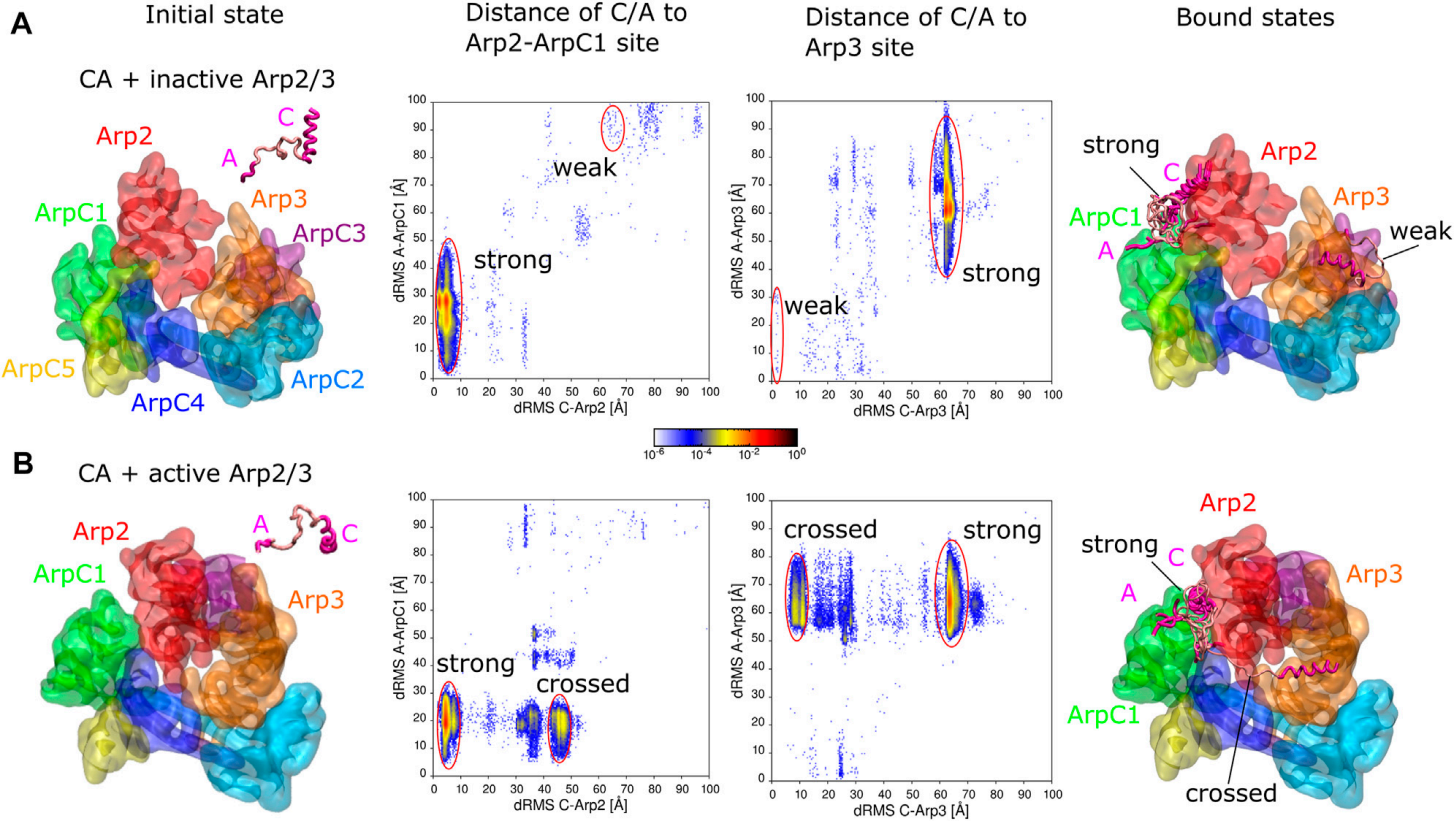
Simulations of CA domain binding to inactive and active Arp2/3 complex. **(A)** Binding of CA to inactive Arp2/3 complex. Left snapshot: initial configuration with CA domain starting separated from Arp2/3 complex. Middle plots: 2D distribution of dRMS values for C and A domains relative to the Arp2-ArpC1 and Arp3 binding sites. dRMS was calculated every 10 time steps. Data binned over .5 × .5 Å^2^. Regions corresponding to strong and weak binding hotspots indicated with red lines. Right snapshot: Bound configurations corresponding to weak and strong hotspots, with a few overlapping examples shown for the strong case. **(B)** Same as panel **(A)**, but for active Arp2/3 complex, with representative bound states shown for the strong and crossed bound states. Both simulations performed in a cubic box of side 300 Å for ∼1.6 *μ*s.

For REMD simulations, 28 temperatures between 180 K and 540 K were used, to explore binding ensembles where protein complexes become bound (unbound) at low (high) temperatures. Temperature swapping was attempted every 100 steps. In REMD simulations the KH-A pairwise interactions strengths were independent of temperature. REMD simulations were run for sufficiently long to sample all candidate bound states, which we checked by repeat of REMD simulations or by checking that replicas span multiple temperatures (Qi et al., 2018). An example of typical dwell time distributions from Figure 1A is shown in Supplementary Figure S1.

All simulations were visualized in Visual Molecular Dynamics (VMD) (Humphrey et al., 1996).

### Structures used

We used two reference conformations for the inactive and active Arp2/3 complex. For the inactive Arp2/3 complex we used the cryo-EM structure of Zimmet et al. (2020) (PDB 6UHC), which is a structure of the human Arp2/3 complex with two bound N-WASP CA domains. We didn’t add any of the missing residues in PDB 6UHC for the inactive complex. We note that a few missing Arp2 residues (M1-G5, L389-R394), especially the N-termini ones, might be involved in actin monomer and C domain binding. The missing residues in Arp3 N- and C-terminal regions can play an inhibitory role for actin monomer binding (Rodnick-Smith et al., 2016). However, since in this complex Arp3 is sterically blocked by Arp2 and not available for actin binding, we didn’t add the missing residues. ArpC1 binds the A domain, however the missing residues are far from that site. Other missing residues include residues in subdomain two of the Arp2/Arp3, as well as residues in ArpC2, ArpC3, ArpC4, and ArpC5; these residues aren’t involved in the binding of daughter filament actin or CA domain, so they were also left missing.

For the active Arp2/3 complex we use the structure of branch junctions obtained by cryo-ET of NIH-3T3 fibroblast cells by Fäßler et al. (2020) (PDB 7AQK). The missing N-terminal residues of Arp2 (K388-R394) were added using MODELLER (Martí-Renom et al., 2000; Webb and Sali, 2016) in a configuration that doesn’t prevent actin monomer binding to its hydrophobic groove. The Arp3 doesn’t have any missing residues in the PDB file. For subunits ArpC1-C5, ArpC5 isn’t expected to be involved in binding to either the mother filament or the daughter filament (Fäßler et al., 2020). Therefore, the N-terminal residues of ArpC5 were left missing. The missing residues in ArpC2 C-terminal region were built for the study of binding to the mother filament, as discussed in the corresponding section of Results. Because this PDB file contains not only the active Arp2/3 complex, but also the mother and daughter actin filaments, the Arp2 and Arp3 are supposed to be in conformations that are ready for actin binding. During the writing of this work, a higher resolution cryo-EM structure of activated Arp2/3 complex from *Bos taurus* was obtained *in vitro* (Ding et al., 2022). The latter structure is overall very similar to that of Fäßler et al. (2020) so we expect the results presented here to hold for both activated structures.

The VCA domain sequence was taken from human WASP (UniprotKB P42768). The V and C helical regions were set as rigid bodies. The V domain configuration (R431-I442) was taken from PDB 2A3Z (Chereau et al., 2005) and the C domain (G465-I481) generated by aligning the WASP sequence to the N-WASP C-helix on the Arp2 or Arp3 binding site of PDB 6UHC (Zimmet et al., 2020). Other residues were assumed flexible.

For actin monomers binding to Arp2 and Arp3 we used the cryo-EM structure of ADP-Pi F-actin [PDB 7K21 from Chou and Pollard (2019)]. The missing N-terminal residues (D1-D3) were added using MODELLER (Martí-Renom et al., 2000; Webb and Sali, 2016). Each actin monomer was set as a rigid body except for D-loop region (H40-S52) that was assumed flexible, similar to Horan et al. (2020).

For mother actin filament, we used the structure from Fäßler et al. (2020) (PDB 7AQK). All eight actin subunits were kept in a rigid configuration relative to each other, but with the D-loops (residues 40–51), many of which are in close proximity to subunits of the Arp2/3 complex, assumed flexible. All the mother filament actin subunits had residues D1-E4 missing. They were left missing during the simulations as they aren’t considered essential for Arp2/3 complex binding. We note that we also explored using the higher resolution F-actin structure from Chou and Pollard (2019) (PDB 7K21) for the mother filament. The F-actin structures in 7K21 and 7AQK are very similar to each other, indicating that Arp2/3 complex does not significantly perturb the mother filament conformation (Fäßler et al., 2020; Ding et al., 2022). However, the Fäßler et al. (2020) structure lead to more stable Arp2/3 complex binding, presumably because even small differences complicate aligning the Arp2/3 complex along the large interface with the mother actin filament.

### Analysis

Distance root mean-square (dRMS) is calculated to quantify the similarity between a simulated conformation with respect to a reference conformation (Kim and Hummer, 2008). Only residue pairs near the binding interface (within 10 Å) of the reference conformation were included for the dRMS calculation. The conformations are considered to be similar to a reference conformation when dRMS is as small as a few Å. The reference conformations are either directly taken from published experimental conformation, or obtained by aligning different components to the experimental conformation using MultiSeq (Roberts et al., 2006) plugin in VMD.

Horan et al. (2020), who used KH-A to study association among actin subunits, found that the dissociation constant (*K*_*d*_) of actin complexes was sensitive to the LJ cutoff when comparing results at three *σ* versus four *σ* cutoff. Larger cutoff values increased complex binding affinity (decreased *K*_*d*_) but favored the most compact configurations, due to the directional nature of long range interactions in these implicit solvent simulations. However, the basic set of binding ensemble configurations was approximately maintained, independently of the three *σ* versus four *σ* cutoff, as well as independently of the temperature, provided it was low enough to keep the association. In this study we don’t aim to derive precise predictions on the absolute or relative magnitude of *K*_*d*_ values, a task largely beyond the limits of the KH model. Thus, similar to Horan et al. (2020), we characterize bound complexes at the lowest REMD temperature of 180 K, where the bound configurations occur with higher probability. Serial simulations were also performed at 180 K. We note that even at 180 K, disordered regions in our simulations don’t collapse into a globule, unlike KH model D which has stronger interactions among solvent accessible residues when compared to KH-A, and has thus been used to model chain collapse (Dignon et al., 2019).

## Results

We used the C_*α*_ model of Kim and Hummer (2008), which has reproduced binding of actin subunits to the barbed and pointed ends of bare and formin-associated actin filaments (Horan et al., 2020; 2018). We represent globular domains as rigid bodies taken from experimentally-determined structures and flexible domains as beads connected by springs. We use simulations to explore binding configurations and interactions between subunits (such as VCA, actin monomer, Arp2/3 complex, actin filament) as they come together, without considering conformational changes within each simulation.

### CA domain distinguishes strong and weak binding sites on Arp2/3 complex

We performed simulations to explore the binding of the CA domain of WASP to the Arp2/3 complex (Figure 1). The binding of CA domain to Arp2/3 complex at two distinct binding sites, the Arp2-ArpC1 site and the Arp3 site, is an important step in its activation (Zimmet et al., 2020).

#### CA binding to inactive Arp2/3 complex

We first tested if KH-A reproduces binding of CA to the inactive Arp2/3 complex. We took the cryo-EM structure of the inactive complex with CA bound to two distinct sites from PDB 6UHC (Zimmet et al., 2020), removed the bound CA domains, and performed REMD simulations with a single CA domain of human WASP (G465 - D502) and inactive Arp2/3 complex, initially separated (Figure 1A). The inactive Arp2/3 complex was fixed as a single rigid body. The C domain (G465- I481) was fixed in a helical conformation as in the Arp2-ArpC1 binding site of PDB 6UHC, whereas the rest of the CA domain was assumed flexible. dRMS distances relative to both the Arp2-ArpC1 site and the Arp3 site were calculated for C and A domains individually using the cryo-EM structure PDB 6UHC as reference (G465—I481 for C and E498—D501 for A).

The CA domain found the Arp2-ArpC1 site as the dominant binding site, indicated by “strong” in Figure 1A, in agreement with Zimmet et al. (2020) who found this site to be the stronger one experimentally. In this binding mode, the C-helix is placed precisely compared to reference structure (less than 10 Å dRMS), whereas the A domain fluctuates around its anticipated binding location relative to ArpC1 (Figure 1A, encircled in red). Thus, there is a large variation in the A-to-ArpC1 dRMS values within the strong hotspot of the Arp2-ArpC1 site dRMS plots. These conformations have C helices well aligned, whereas A domain binds transiently to sites on Arp2 and ArpC1.

The CA domain also found the Arp3 site observed by cryo-EM, though with much smaller probability compared to the strong site, indicating weaker affinity (indicated “weak” as in Zimmet et al. (2020) in Figure 1A). Within this weak hotspot of the Arp3 site dRMS plot, the C region is placed precisely compared to the reference structure while the A domain fluctuates around its anticipated location, similar to the strong case.

Even though the Arp3 site had obviously weaker affinity in our simulations, we cannot precisely estimate the relative affinity with respect to the Arp2 site, both due to the limitations of the CG model (Horan et al., 2020), as well as the requirement of long equilibration times. To demonstrate the statistical properties of these weak states in the simulation, we calculated the 2D distribution of dRMS for the entire simulation and the second half of the simulation for representative temperatures from the lowest (180 K) to the highest (540 K) (Supplementary Figure S2). While the Arp3 site was observed throughout the simulation, weaker bound states appeared as transient hotspots at low temperatures in the early part of the simulation or as weak hotspots at intermediate temperatures in the second half of the simulation. At low temperatures, only the strong hotspot was observed for the second half of the simulation.

Additional bound states corresponding to weak hotspots in Figure 1A are shown in Supplementary Figure S3. Though these aren’t very reliable predictions and have no apparent biological significance, we report them since they may reflect transient configurations.

#### CA binding to active Arp2/3 complex

In the activation mechanism supported by Zimmet et al. (2020), CA remains associated with Arp2/3 complex as Arp2 and Arp3 transition into a short-pitch configuration (corresponding to partial or full activation of the complex) and prior to Arp2/3 complex binding to a mother filament. Thus, to study how CA might bind to the fully active Arp2/3 complex, we performed simulations similar to those with the inactive complex, but replacing the Arp2/3 complex with the cryo-ET structure PDB 7AQK of Fäßler et al. (2020) (Figure 1B). dRMS distances were calculated relative to aligned binding to the Arp2-ArpC1 site and the Arp3 sites. More specifically, as a reference configurations, we aligned Arp2, ArpC1 or Arp3 of the active complex to the inactive complex to obtain the reference conformations of C and A binding sites on the active Arp2/3 complex for the Arp2-ArpC1 site or the Arp3 site, respectively.

Similar to the inactive case, and as expected by the model of Zimmet et al. (2020), the CA domain found a spot close to the Arp2-ArpC1 site as the dominant binding site (“strong” in Figure 1B). In this binding mode however, the C-helix goes deeper into the interface between Arp2 and ArpC1 (which are closer to each other in the active complex), whereas the A domain fluctuates around its anticipated binding location relative to ArpC1 (Figure 1B, red circles).

We didn’t observe any binding of CA to the Arp3 site of the active Arp2/3 complex, possibly because it is in a conformation which isn’t available for CA domain binding. We note that the C terminus of Arp3 isn’t sterically blocking C binding to this site.

The CA domain also bound with high probability to the active Arp2/3 complex in a configuration we call “crossed”, where the C domain binds Arp3 and A domain binds ArpC1. This conformation wasn’t observed for the inactive Arp2/3 complex, in which the Arp3 and ArpC1 are farther from each other and thus CA cann’t reach the two sites simultaneously. The crossed configuration is interesting because it could potentially stabilize the active Arp2/3 conformation. However, in this crossed configuration, the C helix is not optimally oriented for delivery of actin monomers through the V domain.

Additional bound states corresponding to weak hotspots in Figure 1B are shown in Supplementary Figure S3.

#### CA binding to inactive and active Arp2/3 complex with Arp3 site C helix conformation

Another set of simulations of CA binding to active and inactive Arp2/3 were performed, under the same condition as in Figure 1, but with the C domain rigid conformation taken from the Arp3 binding site of PDB 6UHC rather than from the Arp2-ArpC1 site (Supplementary Figure S4). The results were very similar to those of Figure 1, except that binding to the weak site of the inactive complex occurred more frequently.

### Stable binding of actin monomers to active Arp2/3 complex with and without VCA

#### Interaction of active Arp2/3 complex with two actin subunits

**FIGURE 2:**
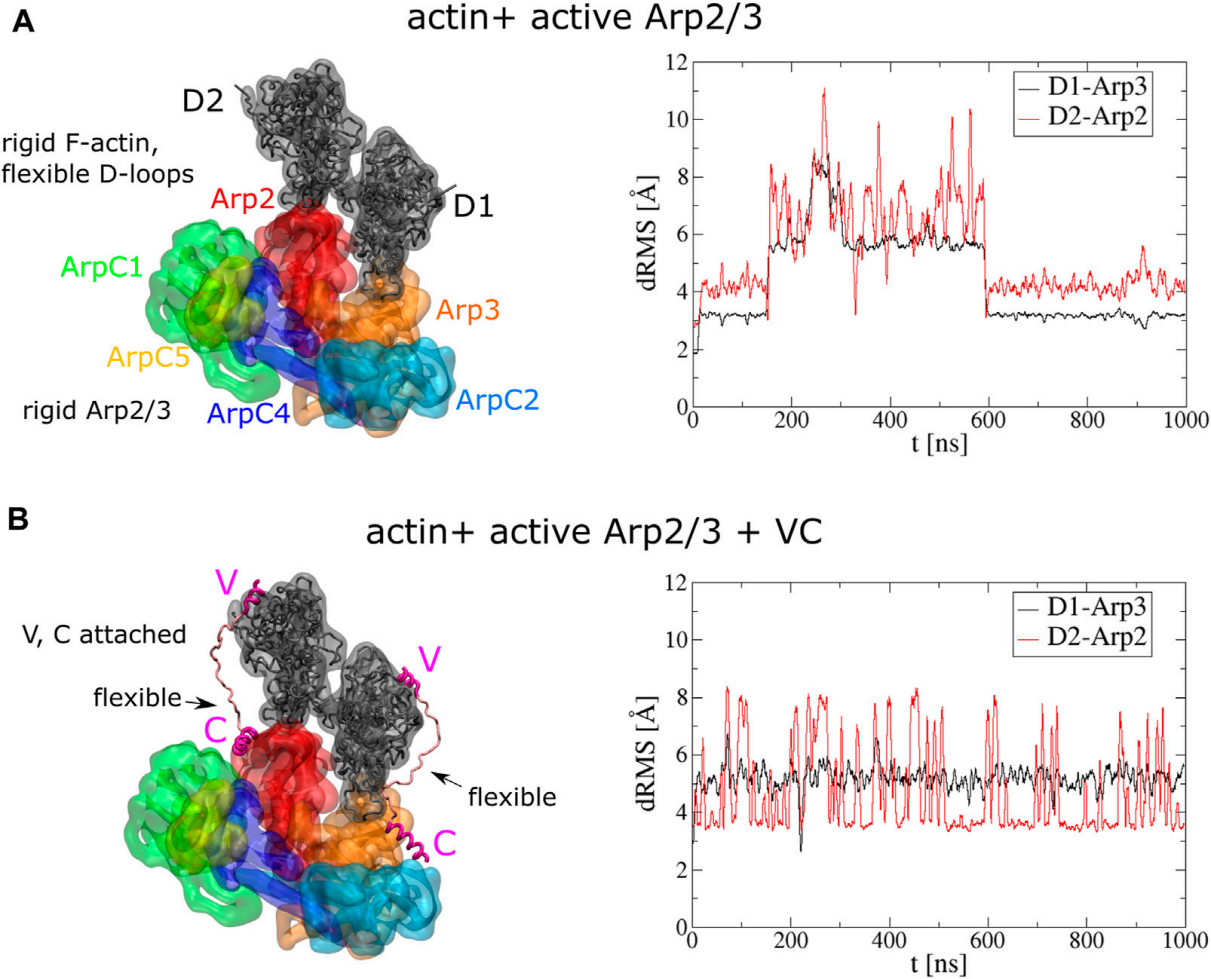
Stable binding of two actin subunits to active Arp2/3 complex. **(A)** Left: Initial conformations of serial simulation starting from two actins bound to Arp2 and Arp3 of active Arp2/3 complex. The ArpC3 subunit isn’t displayed. Right: dRMS of each actin with respect to Arp2 and Arp3 as function of time. **(B)** Left: Initial conformations of serial simulation starting from two actins bound to Arp2 and Arp3 of active Arp2/3 complex. Each actin has a V domain rigidly attached while the C helix is rigidly attached to Arp2/3 complex. Right: dRMS of each actin with respect to Arp2 and Arp3 as function of time. The simulations are performed in cubic boxes of side 300 Å for 1 *μ*s.

We next tested whether Arp2 and Arp3 can stably bind two actin monomers along the direction of the daughter filament, in the presence or absence of VCA domain, using the KH-A model. We thus first performed serial simulations starting with the whole active Arp2/3 complex as a single rigid body and with two separate actin subunits initially placed bound along the long pitch direction to Arp3 (subunit D1 of daughter filament) and Arp2 (subunit D2 of daughter filament), respectively (Figure 2A). These simulations with two actin monomers were performed using the active Arp2/3 complex only, since Arp2 sterically blocks actin binding to Arp3 in the inactive complex. Actin subunits are expected to adopt a flattened F-actin configuration in the daughter filament (Fäßler et al., 2020; Shaaban et al., 2020) so we used the recent high resolution cryo-EM structure of ADP-Pi F-actin (PDB 7K21 from Chou and Pollard (2019)) for the individual actin subunits. Each actin monomer was set as a rigid body except for D-loop region that was assumed flexible, similar to Horan et al. (2020). The simulations started by aligning the actin subunits to the daughter actin subunits of the cryo-ET structure (PDB 7AQK). These initial configurations were also used as the reference configurations for the calculation of dRMS as a function of time to examine the stability of the binding.

During the 1 *μ*s simulation, the two actin subunits remained bound to the active Arp2/3 complex (Supplementary Movie S1). The D-loops of both subunits extended and remained mostly stably into the corresponding hydrophobic grooves of Arp2 and Arp3 along the long pitch direction while the subunits were also attracted to each other along the short pitch direction. Although at 200 ns–600 ns, both subunits show high dRMS value, they later go back to low dRMS conformations. Comparing the two actin subunits, D1 shows more stable binding than D2, as suggested by less fluctuation in the dRMS value. This is because D1 can form short-pitch interaction with both Arp2 and D2, while D2 can form short-pitch interaction only with D1.

To further understand how the VCA domain of NPFs might deliver actin to Arp2/3 complex, and knowing that KH-A keeps the D1 and D2 subunits close to their expected daughter filament configuration, we next run serial simulations with actin and Arp2/3 complex connected by two VC domains (R431-I481) of WASP (Figure 2B). The C domains were fixed rigidly on Arp2 or Arp3 according to the cryo-EM structures of the inactive Arp2/3 complex (Zimmet et al., 2020). The C helix binding sites were obtained by aligning the Arp2 or Arp3 of the active Arp2/3 complex to the inactive complex. The helical region of V domains (R431-I442) were also rigidly attached to the corresponding actin subunits by aligning to a cryo-EM structure with WH2 associated with actin (PDB 2A3Z) (Chereau et al., 2005). The linker between the V and C helix regions was set flexible for each VC domain fragment. Compared to the simulation without VC domains, this simulation shows more stable binding of actins to Arp2/3 complex (Figure 2B, Supplementary Movie S2). The dRMS value doesn’t show drastic change during the simulation. D1 is associated with less fluctuation in dRMS value compared to D2, which is similar to the simulation without VC.

#### Interaction of active Arp2/3 complex with a single actin subunit.

While two actin subunits bind Arp2/3 complex stably when both present (Figure 2A), we also asked if a single actin subunit binds stably. We run similar serial simulations with a single actin subunit, initially bound in a long pitch configuration to Arp2 and Arp3, respectively, of active Arp2/3 complex. The starting position of each actin, as well as the actin monomer model with a flexible D-loop, was the same as the simulation with two bound subunits. Again, these simulations were performed both with and without VC domains. Either with or without VC domain, the single actin subunits fluctuate further from their initial position as compared to the two subunits case, over the same time, for both D1 and D2 (Supplementary Figures S5, S6, Supplementary Movies S3–S6).

In the simulations without the VC domain, D1 and D2 tilt away from their initial F-actin reference location towards lower energy states, as evident by plots of increasing dRMS and lower energy over time (Supplementary Figure S5). D1 rotated in a direction that keeps the D-loop short-pitch interaction to Arp2, but subdomain four unbinds Arp3, making a tilted configuration (similar to BE2 state of F-actin monomer in Horan et al. (2020) (Supplementary Movie S3). D2 rotated in a direction that brought subdomain four close to ArpC1, resulting in a larger dRMS compared to D1 (Supplementary Movie S4). We note that the single actin fluctuations may be overestimated, given that the KH-A model appears to underestimate the strength of long-pitch contacts involving subdomain four in the actin filament Horan et al. (2020).

In simulations where single actin subunits were connected to Arp2/3 complex with a VC domain, rigidly attached to each as in Figure 2, we also observed a tilt of both D1 and D2 away from their initial F-actin-like placement toward configurations with lower energy (Supplementary Figure S6). D1 and D2 actin subunits both rotated about the D-loops in the same direction as they do in the early stage of the simulation without the VC domain (Supplementary Movies S5, S6). However, the actin subunits had less freedom to move when constrained by the VC domains.

### Interaction of actin and VCA with inactive Arp2/3 complex

#### Actin binding to Arp2 from the bulk or delivered through the VCA domain

Knowing that the coarse grained model keeps actin close to Arp2 and Arp3 of the active complex, we next performed simulations to see how such bound conformation can be obtained. In the mechanism of Zimmet et al. (2020), actin bound to V domain is first delivered to the Arp2 site of the inactive complex. Therefore we switched to the inactive Arp2/3 complex to study how actin can be delivered to inactive Arp2, either by directly binding to Arp2 or else delivered to it after CA domain first binds to the Arp2-ArpC1 site.

**FIGURE 3:**
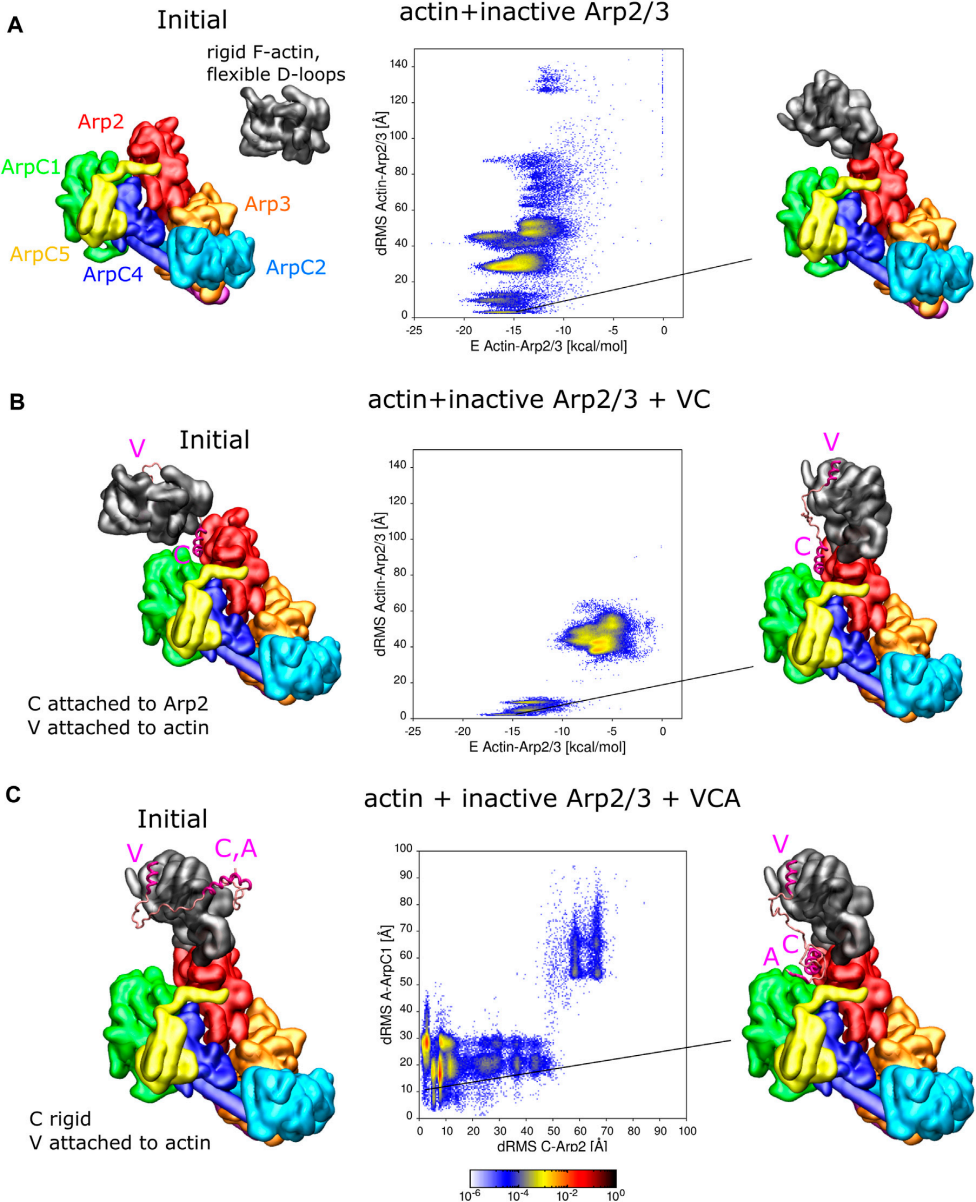
Interaction of actin and VCA with inactive Arp2/3 complex. **(A)** REMD simulations of actin delivery to Arp2 of inactive Arp2/3 complex. The actin monomer is in the rigid F-actin conformation with flexible D-loop. Left: Initial conformation with actin separated from Arp2/3 complex. Middle: 2D-distributions of dRMS vs. binding energy are calculated and plotted in bins of .1 kCal/mol × .5 Å. Right: Example of conformation are randomly selected from the lowest dRMS hotspot. **(B)** Same as panel A but when actin and Arp2/3 are connected by the VC domain, with V rigidly attached to actin and C domain rigidly attached to the Arp2-ArpC1 site. **(C)** REMD simulations of CA binding to Arp2/3 complex with V rigidly fixed on actin. The actin monomer is kept fixed in a long pitch configuration. The C helix is kept rigid [in same configuration as panel **(B)**] and allowed to move with the rest of flexible CA region. Left: Initial conformation with CA separated from Arp2/3 complex. Middle: 2D dRMS distributions of A and C relative to their binding sites on ArpC1 and Arp2, respectively, in bins of .5 × .5 Å. Right: Example from indicated location in dRMS plot. The simulations were performed in cubic boxes of side 300 Å for **(A)** 780 ns, **(B)** 1.2 *μ*s, and **(C)** 1.8 *μ*s.

Two sets of REMD simulations were set up with actin and inactive Arp2/3 complex initially unbound. One has the actin and Arp2/3 complex completely separated (Figure 3A). The other has the actin attached to Arp2 through the VC domain fragment as the serial simulation of Figure 2, whereas the actin is initially not bound to the Arp2/3 complex (Figure 3B). We used the same F-actin conformation and D-loop flexibility as in Figure 2 (so we don’t consider the effects of twisted G-actin versus flattened F-actin that have some influence on actin monomer binding to the barbed end of actin filaments, even in the KH-A model (Horan et al., 2020). The reference conformation for dRMS calculation was obtained by aligning the Arp2 of the inactive Arp2/3 complex to the Arp2 of the active complex (PDB 7AQK), and then aligning the corresponding D2 actin. For both cases we plotted dRMS vs. binding energy between the actin subunit and Arp2/3 complex.

For the simulation without VC domain, the actin forms a series of complexes with the Arp2/3 complex (Figure 3A). There are multiple hotspots with comparable low binding energy, including delivery to Arp2 among these conformations. The most populated hotspot corresponds to actin bound to both Arp2 and ArpC1 through a different interface, where more residues are involved.

For the simulation where actin is attached to the Arp2/3 complex by the VC domain, complex formation is limited to two regions in the dRMS vs. energy plot, due to the limited length of the VC domain. The actin subunit binds the Arp2/3 complex in a daughter filament orientation (low dRMS hotspots) and an incorrect orientation (high dRMS hotspots). The filament orientation binding corresponds to drastically lower energy compared to the other region, indicating the stability of the binding. The incorrect orientation, although associated with weaker binding between actin and the Arp2/3 complex, has higher probability; this is due to the linker region which tangles and interacts with itself or interacts with the Arp2/3 complex, lowering of the total energy.

#### Association of CA with inactive Arp2/3 complex when V-actin is bound to Arp2

Figure 3B showed one possible pathway of VCA-actin binding to inactive Arp2/3, where CA binds first to the Arp2-ArpC1 site, followed by V-actin delivery to Arp2. An alternative pathway is for V-actin to first associate with Arp2, followed by the binding of C and A regions to their Arp2-ArpC1 site. To examine this possibility, we placed a rigid F-actin with associated rigid V domain on the D2 site used as a reference in Figures 3A, B and now allowed the CA region to explore space, in REMD simulations (Figure 3C). In these simulations, the C helix was kept rigid in its Arp2-ArpC1 configuration, as in Figures 3A, B, Interestingly, the results of Figure 3C show that the CA helix associated close to the Arp2-ArpC1 site with high probability, similar to free CA in Figure 1A. The C helix came frequently close to the reference site, while the A domain fluctuated near ArpC1.

#### Binding of VCA-actin to inactive Arp2/3 complex

In addition to the simulations of Figure 3 where either actin or C were fixed on Arp2/3 complex, we performed REMD simulations with VCA-actin initially completely separated from the inactive Arp2/3 complex (Supplementary Figure S7). The VCA domain was attached to an F-actin with flexible D-loop through the V domain, the C domain was set rigid as in Figure 3C, and the remaining residues assumed flexible. We found that CA finds the strong spot on Arp2-ArpC1 with high probability, similar to Figure 1, with a defined C binding site and a fluctuating A domain. Actin also binds Arp2 in a long-pitch-like configuration with high probability, similar to Figure 3A. However, actin and the C domain didn’t bind simultaneously to their reference configurations, unlike the case in Figures 3B, C where either one of them was fixed on Arp2. While this result may indicate a competition between CA and D-loop for the region near the Arp2 hydrophobic groove, it’s also very likely that this observation reveals a limitation of the coarse-grained model to precisely place C as well as the D-loop on nearby sites on Arp2 in a way that they don’t block each other. The few N and C terminal residues on Arp2, that weren’t included in this simulation, are also located near this interface and may also play an additional role. The simulation was repeated four times to sample more possible conformations. However, none of the simulations showed C-helix and actin correctly binding simultaneously.

### Actin delivery to active Arp2/3 complex

**FIGURE 4:**
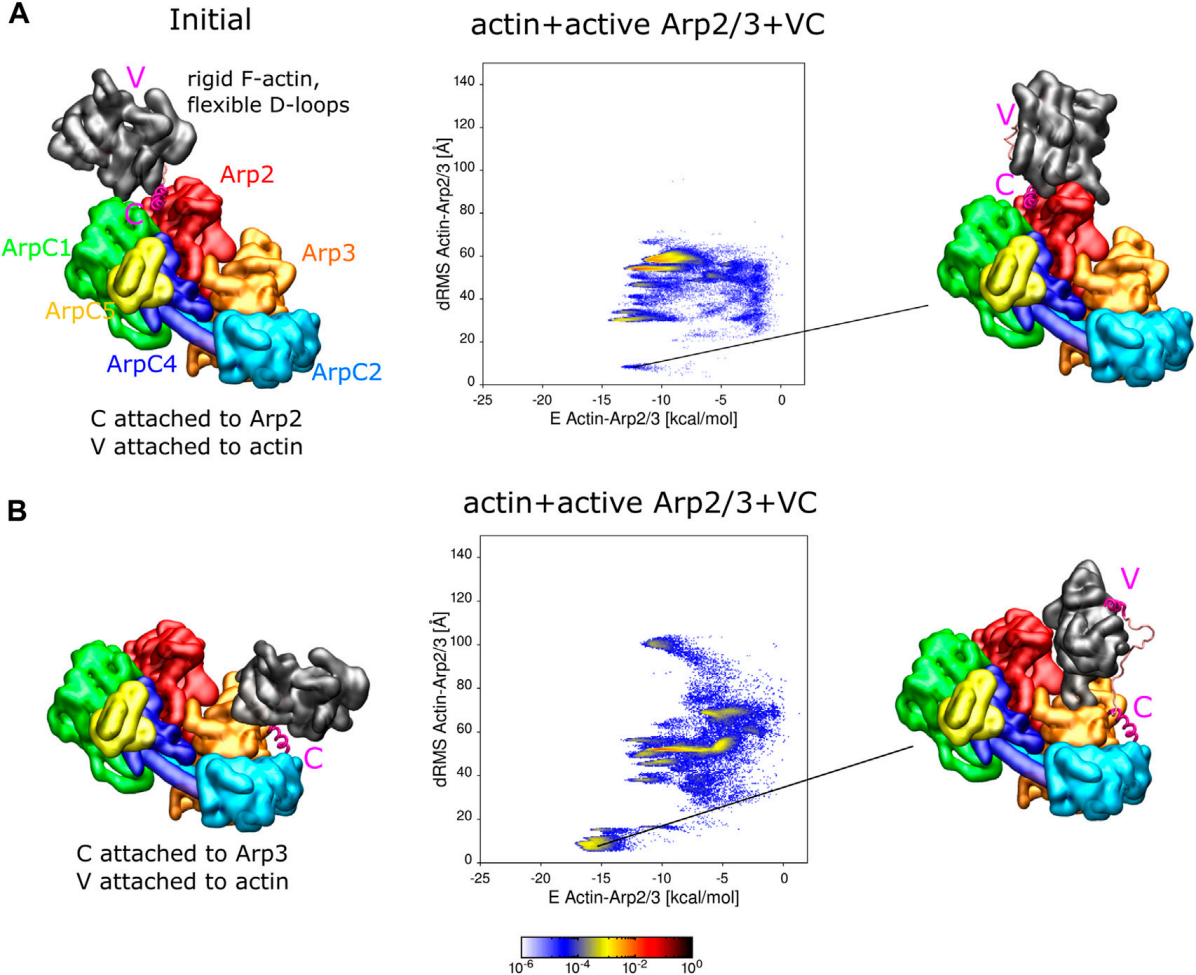
Actin binding to active Arp2/3 complex with attached VC domain. **(A)** REMD simulations of actin delivery to Arp2 of inactive Arp2/3 complex. Actin and Arp2/3 complex are connected by the VC domain, with V rigidly attached to actin and C domain rigidly attached to the Arp2-ArpC1 site. The actin monomer is in the rigid F-actin conformation with flexible D-loop. Left: Initial conformation with actin separated from Arp2/3 complex. Middle: 2D-distributions of dRMS vs. binding energy are calculated and plotted in bins of .1 kCal/mol × .5 Å. Right: Example of conformation are randomly selected from the lowest dRMS hotspot. **(B)** Same as panel **(A)** but with C domain attached to the Arp3 site. Simulations performed in cubic boxes of sides 300 Å for **(A)** ∼1.7μ s, and **(B)** ∼950 ns.

We also performed simulations to study the delivery of actin to Arp2 and Arp3 of the active Arp2/3 complex, where both Arp2 and Arp3 sites (i.e., D1 and D2 subunit locations) are available (Figure 4). We examined the delivery of actin by REMD simulations. We fixed the C domain to either binding site on a rigid Arp2/3 complex, as in Figure 2B. The V domain was rigidly attached to the actin subunit in the F-actin configuration with flexible D-loop as in Figure 2B. The reference configurations for the dRMS calculation were the same as in Figure 2B for each of the simulations.

We found that the actin subunits bind to Arp2/3 complex in a few different conformations, which include low energy states with actin placed very close to the anticipated D1 or D2 locations (low dRMS hotspots in Figure 4). When C is attached to Arp2, the actin subunit can form various bound states with Arp2/3 complex, including close to the reference configuration at the D2 site (Figure 4A). The probability of finding the actin subunit at the D2 location was smaller, and the energy between actin and Arp2/3 complex higher, than in the corresponding simulations with the inactive complex of Figure 3B. However, the different resolution and different missing residues between the active and inactive complexes, as well slow simulation equilibration time prevents us from reaching definite conclusions regarding differential affinities for actin or VCA with the active or inactive Arp2/3 complex. When C is attached to Arp3, the actin subunit mainly formed two types of complexes with the Arp2/3 complex, with the one close to the D1 reference having the higher probability and lower energy between actin and Arp2/3 complex (Figure 4B).

In simulations with single free actin monomers interacting with Arp2/3 complex, the actin monomers were also found to associate close to the Arp2 and Arp3 sites (Supplementary Figure S8).

### Arp2/3 complex binding to mother actin filament

**FIGURE 5:**
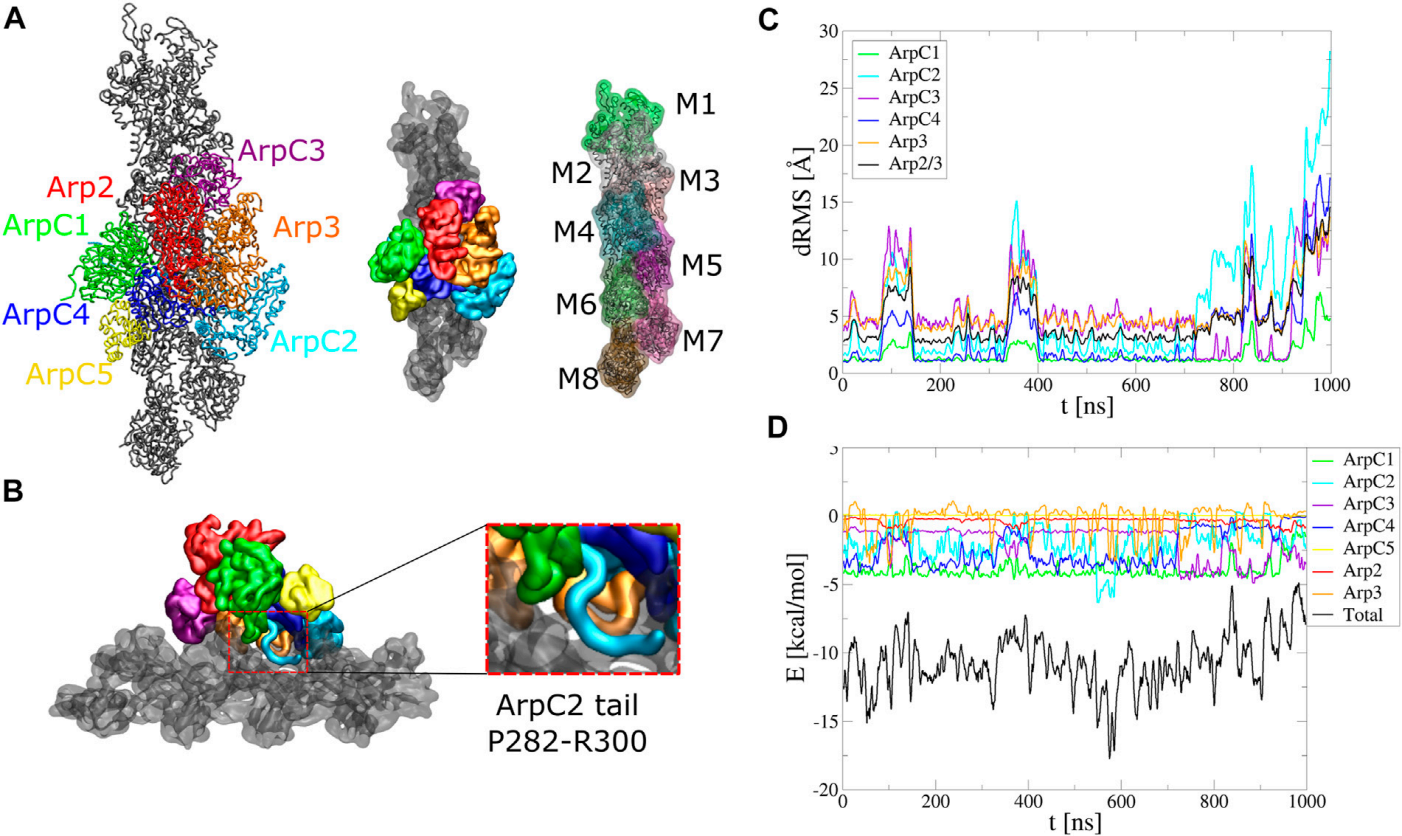
Arp2/3 complex binding to mother actin filament. Serial simulation starting from active Arp2/3 complex bound to the mother filament (PDB 7AQK). The Arp2/3 complex and mother actin filaments are treated as separate rigid bodies except for flexible regions in actin, Arp2, Arp3, and ArpC2 tail. **(A)** Initial configuration. **(B)** Side view showing typical configuration of ArpC2 tail during the simulation. Enlarged region shows the ArpC1 protrusion loop and ArpC2 C-terminal tail built between ArpC1 and ArpC4. **(C)** dRMS between mother actin filament and each of Arp2/3 complex subunits, as well as with entire Arp2/3 complex, with respect to PDB 7AQK. Arp2 and ArpC5 don’t start close enough to the actin filament to define a dRMS according to the criteria used in this paper. **(D)** Interaction energies between mother actin filament and Arp2/3 complex subunits or entire Arp2/3 complex as a function of time. The simulation was performed in a cubic box of side 300 Å for 1 *μ*s.

Given the success of reproducing actin and CA delivery to Arp2/3 complex, we wondered if the KH-A model can also be used to study the binding of active Arp2/3 complex to mother actin filaments. We thus performed serial simulations to study these interactions (Figure 5A). In these simulations, we used a rigid active Arp2/3 complex from Fäßler et al. (2020) (PDB 7AQK), as in Figures 1, 2, 4, but we allowed the regions of subdomain two of Arp2 and Arp3 that correspond to the D-loop of actin (40–53 for Arp2 and 45–58 of Arp3), which can potentially interact with the mother actin filament, to be flexible. For the mother filament, all eight actin subunits were kept in a rigid configuration relative to each other, but with the D-loops (residues 40–51), many of which are in close proximity to subunits of the Arp2/3 complex, assumed flexible (see Methods).

Both structures with Arp2/3 complex bound to actin filaments (Fäßler et al., 2020; Ding et al., 2022) lack the C-terminal tail region of ArpC2, likely a flexible region. In simulations where Arp2/3 complex was started with the ArpC2 tail missing, the complex progressively tilted away from the mother filament, opening a gap around the location of ArpC2 tail (Supplementary Figure S9). We thus added the ArpC2 C-terminal tail as a flexible region between the ArpC1 protrusion helix and ArpC4 and set it as a flexible region (Figure 5B).

With the added ArpC2 tail, the binding was stable and the system remained at low energies for the first 800 ns, after which the complex tilted as it diffused towards higher binding energies (Figure 5C, D, Supplementary Movie S7). The binding energy reflects the contribution of each subunit. In agreement with studies showing the importance of the ArpC1 protrusion helix in Arp2/3 complex activation (Ding et al., 2022), ArpC1 is the subunit that contributes the most. This is followed by ArpC2 and ArpC4, subunits known for their role in mother actin filament binding. The flexible tail of ArpC2 interacted with the region between subdomain one and two of F-actin subunit M6, in a typical configuration shown in the inset of Figure 5B. ArpC3 bound to the filament more stably over time, with weaker energies compared to ArpC1, ArpC2, and ArpC4. Arp2 and ArpC5 weren’t involved in the binding to the mother filament according to our criteria.

We note that the missing ArpC2 C-terminal tail can lie on either side of the ArpC1 protrusion helix. In Figure 5B we started with the ArpC2 tail between ArpC1 and ArpC5. We also run simulations with ArpC2 C-terminal region built between ArpC1 and ArpC3 (Supplementary Figure S10, Supplementary Movie S8). However, in this case the interaction energy between ArpC2 and the mother actin filament started much weaker and binding of Arp2/3 complex became unstable within 300 ns The ArpC2 tail unbound from the filament quickly and went through a gap between Arp2 and Arp3. Our simulations thus suggest that the ArpC2 tail localizes between the ArpC1 protrusion loop and ArpC5.

We also performed REMD simulations to see if active Arp2/3 complex will bind to the sides of an actin filament when starting from a separate state (Supplementary Figure S11). In order to sample the possible binding conformations efficiently, we reduced the LAMMPS mass variable of the Arp2/3 complex by a factor of 100, which allows faster diffusion of fully rigid objects (Horan et al., 2020). Therefore, the whole Arp2/3 complex was set as a rigid body and the ArpC2 tail was left missing. Preliminary simulations showed high affinity between the Arp2/3 complex and the ends of the actin filament, as well as between the Arp2/Arp3 interface and the actin filament, so we capped these ends with extra large beads (Supplementary Figure S11, magenta spheres). Even though many possible binding conformations have been avoided by these repulsive capping beads, the Arp2/3 complex formed a series of low energy complexes that differed compared to the expected side binding configuration (hotspots with dRMS tens of Å away from the 7AQK reference in Supplementary Figure S11). These configurations had stronger (lower) binding energies compared to ∼−8 kCal/mol when ArpC2 tail was absent (Supplementary Figure S9) and thus occurred with higher probability. It’s possible that these configurations correspond to the strongly bound “dead-end” complexes that don’t lead to daughter branches in prior single molecule imaging of Arp2/3 complex binding to actin filament (Smith et al., 2013).

## Discussion

In this paper we performed coarse-grained molecular dynamics simulations using the KH-A model to simulate steps of Arp2/3 complex activation and nucleation of actin an filament branch. We now review the three questions we raised in the Introduction.

### Does the KH-A model capture known interactions involved in Arp2/3 complex activation?

Despite the simplifications involved in the CG approach, and without any modification of the interaction potentials, the KH-A model successfully reproduced the binding of the C region to the inactive Arp2/3 complex (Figure 1A). The stronger affinity of CA to the Arp2-ArpC1 site is also in agreement with experimental measurements of *K*_*d*_ values for the two binding sites (Zimmet et al., 2020). The model showed that upon C helix binding, the A domain fluctuated around the inactive complex, consistent with the lack of electron density for most of the A region in the cryo-EM structure of Zimmet et al. (2020), though the last four A domain residues weren’t strongly tethered to ArpC1, possibly reflecting limitations of the CG model.

The model also reproduced stable binding of two F-actin subunits to Arp2 and Arp3 of the activated complex (Figure 2A). Single actin subunits also bound stably to Arp2 and Arp3 (Supplementary Figure S5), though they exhibited larger fluctuations compared to the simulation with two actin subunits, which stabilized each other through short-pitch helix interactions. The D-loop of subdomain two held the fluctuating actin attached to Arp2 and Arp3, while subdomain four was less stably attached. We note however that the strength of the long-pitch interaction involving subdomain four might be underestimated in the KH-A model of an actin filament (Horan et al., 2020).

Serial simulations also kept the active Arp2/3 complex bound to the mother actin filament (Figure 5). This observation provided estimates of the predicted contribution to the binding free energy of each subunit of Arp2/3 complex. Interestingly, Arp3 interacted with the filament both attractively and repulsively with the mother filament, as its flexible loop (the loop between *β*4 and *β*5 corresponding to actin’s D-loop) exhibited fluctuating interactions with subunit M5 and the fluctuating D-loop of subunit M6. This observation may help explain why this loop play a dampening role in branch formation (Liu et al., 2013). The added ArpC2 C-terminal tail, though missing from experimental conformations (Fäßler et al., 2020; Ding et al., 2022), was important to stabilize this binding. Presumably, this region was not detected experimentally due to sampling of various conformations and extended linear shape, as in our CG simulations. Prior studies showed that antibodies for the ArpC2 C-terminal region inhibited side-branching (Bailly et al., 2001).

### What do our simulation show about complexes expected to form in the activation model of Zimmet et al. (2020)?

We focus on the mechanism supported by Zimmet et al. (2020), being one of the most recent models based on structural data, noting that it combines data and mechanism from extensive prior works, and that some aspects may be different for the yeast Arp2/3 complex. According to this model, the first step is the delivery of actin to Arp2 along with binding of CA to the Arp2/ArpC1 site. This is a pathway that we essentially reproduced in our simulations. When we attached C to inactive Arp2/3 complex, V-bound actin could be delivered to Arp2 (Figure 3B) and *vice versa*, when V-actin was attached to Arp2, CA could bind close to the Arp2/ArpC1 site (Figure 3C). However, in simulations where both V-actin and CA started detached from the inactive Arp2/3 complex, we only observed either C or actin close to their respective binding sites, but not both together. Precise placement of the C helix and the flexible actin D-loop near the same spot on Arp2 in a way that they don’t block each other might depend on atomistic-level features, beyond the abilities of the coarse-grained model; this may be the reason why such a structure wasn’t observed in these simulations.

The subsequent activation step is the binding of VCA-actin in a long pitch to Arp3 (Zimmet et al., 2020). This step is enabled by the binding of VCA-actin to Arp2/ArpC1, which is expected to induce (or make more frequent) a conformational change bringing Arp2 and Arp3 into a short-pitch helix. Our simulations showed that such delivery of actin to Arp3 is possible: V-actin was delivered when C was attached to the Arp3 site of the active complex (Figure 4B). Our simulations also suggest that the proposed final complex of activated Arp2/3 complex with two bound actin subunits and two VCA domains is a viable possibility (Figure 2B).

In cells, the above VCA-mediated activation processes should occur close to membrane regions with bound dimers or high concentrations of VCA-containing proteins. Presumably, free actin and profilin-actin can bind to Arp2 of the inactive Arp2/3 complex in the cytoplasm (Figure 3A), but should be unable to activate it in the absence of VCA.

### What do our results suggest for future work?

After binding of the complex with two VCA-actin subunits to the side of a mother actin filament, the final step is detachment of VCA from the Arp2/3 complex and growth of the daughter filament by polymerization. We note some observations in our simulations that may relate to VCA detachment. When comparing the inactive and active cases, the binding site of the C helix to the active complex was slightly displaced compared to the Arp2 site of the active complex (lying in between Arp2 and ArpC1 Figure 1). The delivery of V-actin to the Arp2 site of the active complex (Figure 4A) also occurred with lower probability when compared to the inactive complex (Figure 3B). These observations could reflect internal strains that develop upon completion of Arp2/3 complex activation, promoting VCA release.

While we reproduced C helix binding to the Arp3 site of the inactive complex, we didn’t observe the same for the active complex (Figure 1). Given the weakness of the inactive complex interaction however, it’s unclear if this might also indicate a tendency for C detachment. We note that in the yeast Arp2/3 complex, the affinity of CA for that site increases 40-fold upon binding to the mother filament (Ti et al., 2011).

Our simulations also showed structures that may reflect non-specific complexes, such as a crossed binding mode of CA domain to the active Arp2/3 complex. This binding may potentially pull Arp3 and ArpC1 close to each other, pushing the complex towards an active configuration in a non-canonical way. To our knowledge, there have been no experimental results supporting this hypothesis so far.

The KH-A coarse-grained model as employed here required the folded domains of proteins to be set as rigid. The transition between the inactive and active conformations cann’t be directly studied with this method. CG-MD simulations with added flexibility or higher resolution that can capture protein backbone motions, followed by all-atom MD simulations informed by CG models, should help to study this essential transition.

## Data Availability

Input LAMMPS files allowing reproduction of our results are available at https://github.com/shz720/Arp23_CG.
