# Supplementary material for: Steps of actin filament branch formation by Arp2/3 complex investigated with coarse-grained molecular dynamics
Source: Front Cell Dev Biol. 2023 Jan 17;11:1071977. doi: 10.3389/fcell.2023.1071977 (PMC9887124; doi:10.3389/fcell.2023.1071977)
Supplement: Supplementary file 3 [file DataSheet1.pdf]

Supplementary Material: Steps of actin filament branch formation by Arp2/3 complex investigated with coarse-grained molecular dynamics

Shuting Zhang and Dimitrios Vavylonis

SUPPLEMENTARY MOVIES

Movie 1 Serial simulation of two actin subunits bound to active Arp2/3 complex without VC domain (Figure 2A). The simulation was performed in a cubic box of side 300 Å for 1μs.

Movie 2 Serial simulation of two actin subunits bound to active Arp2/3 complex at presence of VC domain (Figure 2B). The simulation was performed in a cubic box of side 300 Å for 1μs.

Movie 3 Serial simulation of a single actin subunit bound to the Arp3 of active Arp2/3 complex without VC domain (Figure S5A). The simulation was performed in a cubic box of side 300 Å for 1μs.

Movie 4 Serial simulation of a single actin subunit bound to the Arp2 of active Arp2/3 complex without VC domain (Figure S5B). The simulation was performed in a cubic box of side 300 Å for 1μs.

Movie 5 Serial simulation of a single actin subunit bound to the Arp3 of active Arp2/3 complex in the presence of VC domain (Figure S6A). The simulation was performed in a cubic box of side 300 Å for 1μs.

Movie 6 Serial simulation of a single actin subunit bound to the Arp2 of active Arp2/3 complex in the presence of VC domain (Figure S6B). The simulation was performed in a cubic box of side 300 Å for 1μs.

Movie 7 Serial simulation of the active Arp2/3 complex bound to the mother actin filament, with ArpC2 C-terminal tail built between ArpC1 protrusion loop and ArpC4 (Figure 5). The simulation was performed in a cubic box of side 400 Å for 1μs.

Movie 8 Serial simulation of the active Arp2/3 complex bound to the mother actin filament, with ArpC2 C-terminal tail built between ArpC1 protrusion loop and Arp3 (Figure S10). The simulation was performed in a cubic box of side 400 Å for 1μs.

SUPPLEMENTARY FIGURES

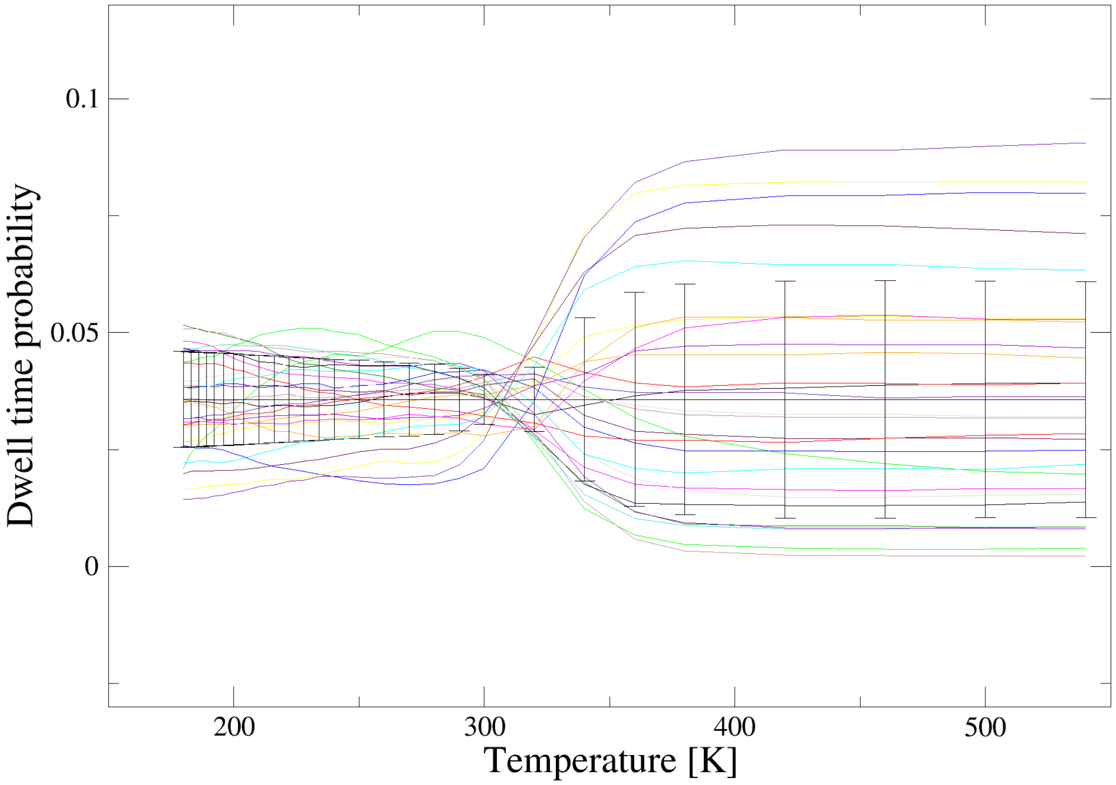

Figure S1. Replica dwell time distribution of the REMD simulation of CA domain and inactive Arp2/3 complex of Figure 1A. Each line shows the distribution of time on different temperatures over the 1.6 μs simulation. The black line is the average of all 28 replicas. The error bars are standard deviations. Replicas span multiple temperatures, indicating the system is approaching equilibrium where the time a replica spends at every temperature site should be similar.

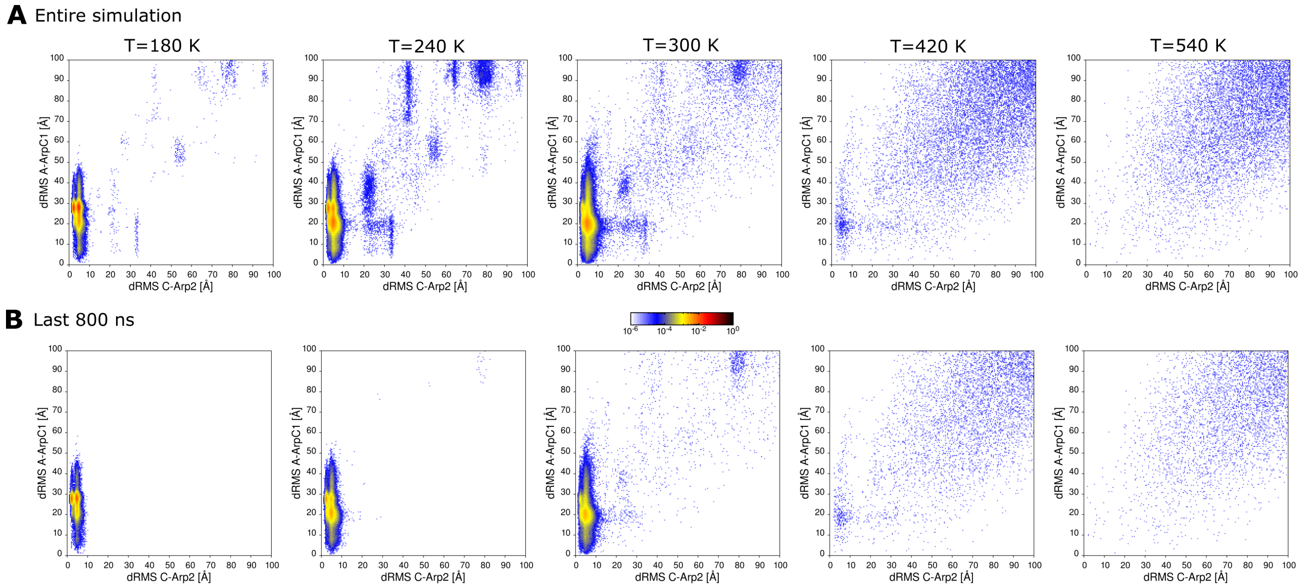

Figure S2. Temperature- and time-dependence of the simulation with CA domain and inactive Arp2/3 complex of Figure 1A. 2D distributions of dRMS of C domain to Arp2 binding site and A domain to ArpC1 binding site, as in Figure 1A for the (A) entire simulation, and (B) the last 800 ns (second half of the simulation). At 540 K, the CA domain is essentially always dissociated from the Arp2/3 complex through the entire simulation. The system gets trapped at the lowest energy hotspot in the second half of the simulation at low temperatures (180 K-240 K in panel B). Weaker bound states are observed as transient hotspots at low temperatures in the early part of the simulation (180 K-300 K of panel A) or as weaker hotspots at intermediate temperatures in the second half of the simulation (300 K-420 K of panel B).

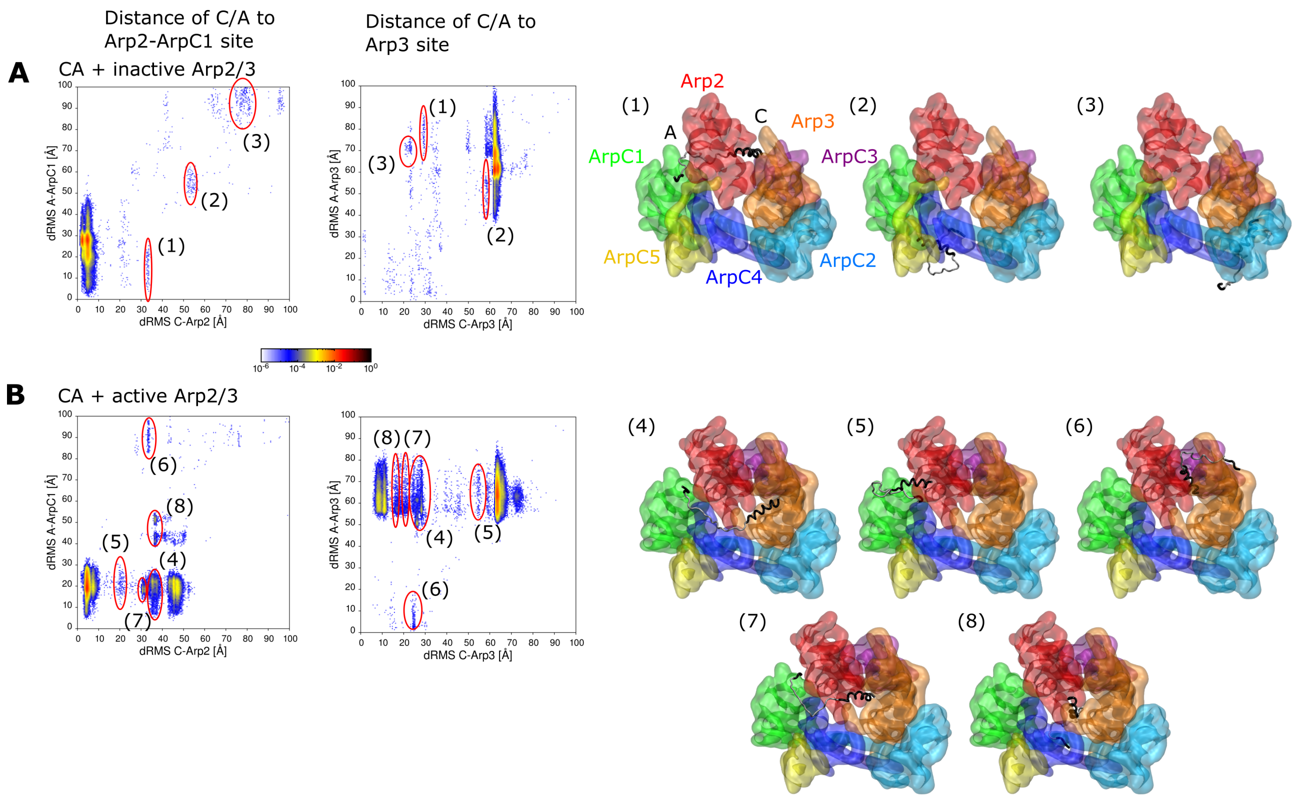

Figure S3. Additional conformations from simulations of CA domain binding to inactive and active Arp2/3 complex. This figure shows conformations beyond those of Figure 1. **(A)** Binding of CA to inactive Arp2/3 complex. Left plots: 2D distribution of dRMS values for C and A domains relative to the Arp2-ArpC1 and Arp3 binding sites that are the same as Figure 1A. Right snapshots (1)-(3): Conformations of CA domain binding to inactive Arp2/3 complex observed in the simulation in three main weak hotspots marked with red circles in the plots. **(B)** Same as panel A, but for active Arp2/3 complex, with five snapshots ((4)-(8)) displayed. Simulations performed in cubic boxes of side 300 Å for $\sim 1.6\ \mu$s.

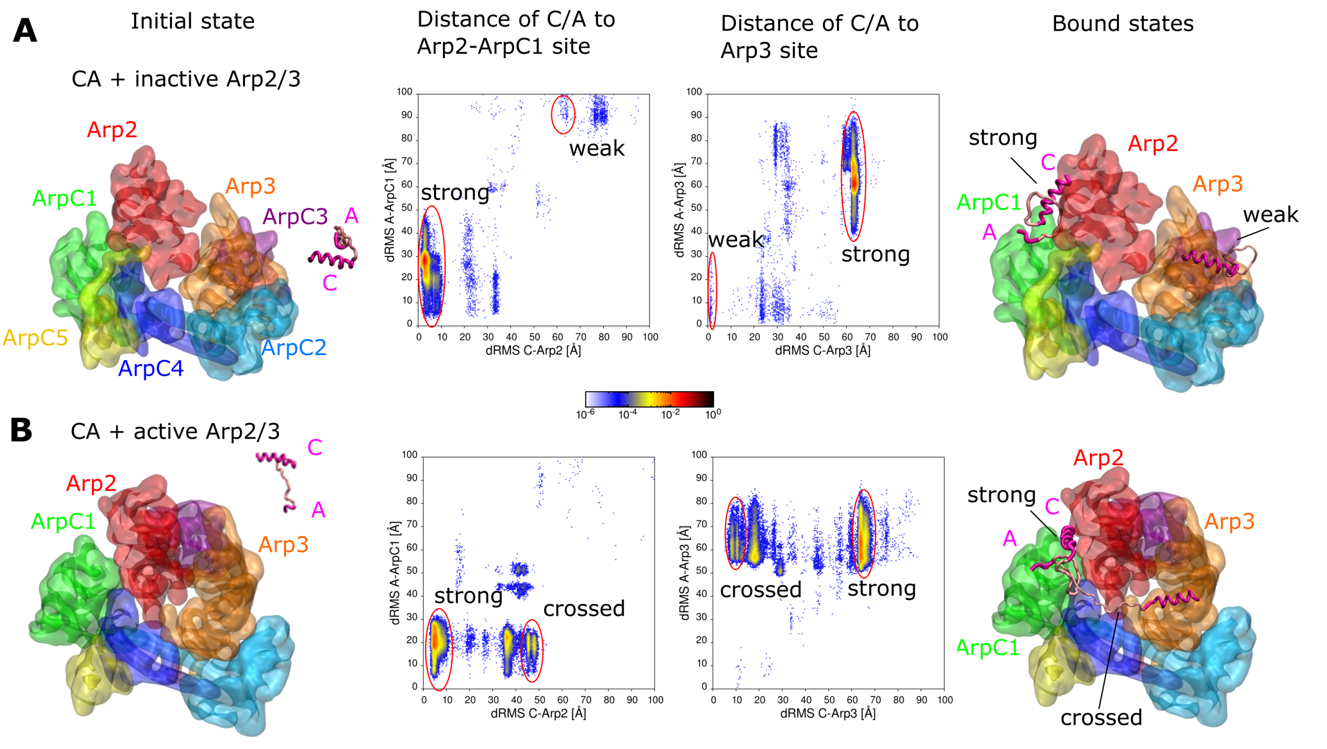

Figure S4. Simulations of CA domain binding to inactive and active Arp2/3 complex using alternate C helix conformation. Same as Figure 1 but C domain (G465-I481) fixed rigid as in C domain bound to Arp3 site of PDB 6UHC instead of the Arp2-ArpC1 site as in Figure 1. **(A)** Binding of CA to inactive Arp2/3 complex. Left snapshot: initial configuration with CA domain starting separated from Arp2/3 complex. Middle plots: 2D distribution of dRMS values for C and A domains relative to the Arp2-ArpC1 and Arp3 binding sites. Data binned over 0.5×0.5 Å^2. Regions corresponding to strong and weak binding hotspots indicated with red lines. Right snapshot: Bound configurations corresponding to weak and strong hotspots, with a few overlapping examples shown for the strong case. **(B)** Same as panel A, but for active Arp2/3 complex, with representative bound states shown for the strong and crossed bound states. Simulations performed in cubic boxes of side 300 Å for $\sim 1.6\ \mu$s.

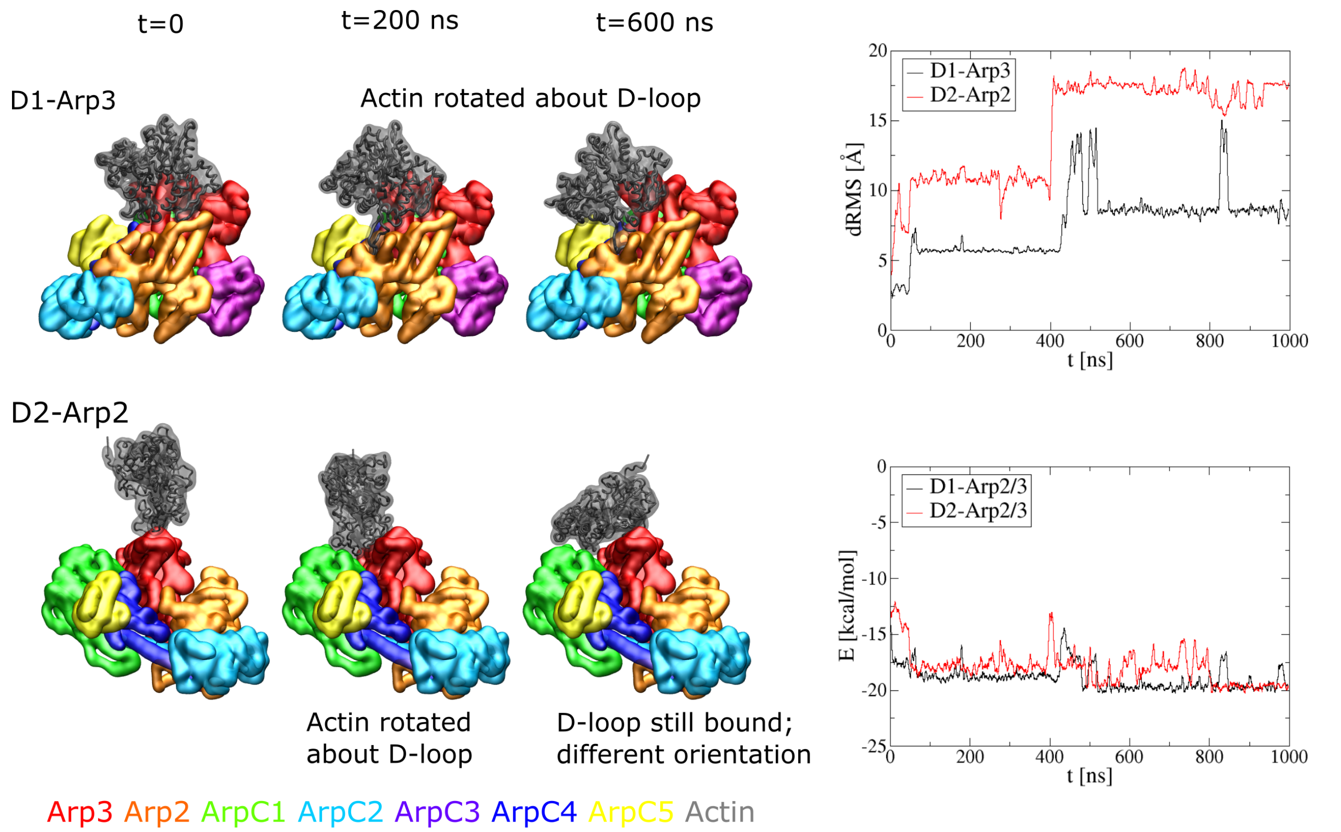

Figure S5. Single actin bound to Arp2 or Arp3 of the active Arp2/3 complex without VC domain. Serial simulation of a single actin bound in a long pitch configuration to Arp3 (top) or Arp2 (bottom) of the active Arp2/3 complex. The serial simulations start from the reference conformations of Figure 2. The dRMS and binding energy are plotted as functions of time for each simulation between the actin and the Arp2/3 complex. Representative conformations are displayed. Simulations performed in cubic boxes of side 300 Å for 1 μs.

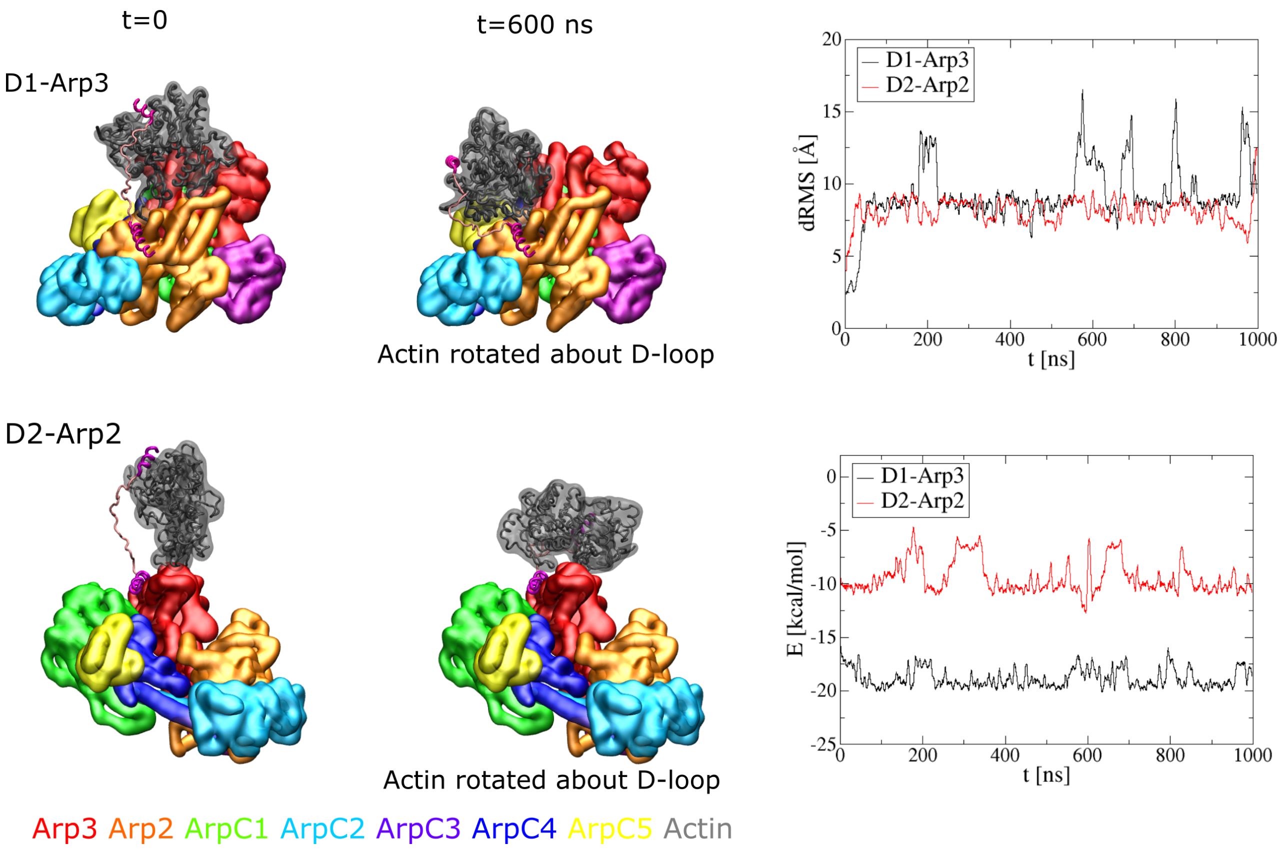

Figure S6. Single actin bound to Arp2 or Arp3 of the active Arp2/3 complex in the presence of VC domain. Serial simulation of a single actin bound in a long pitch configuration to Arp3 (top) or Arp2 (bottom) of the active Arp2/3 complex at presence of VC domain. The serial simulations start from the reference conformation of Figure 2. The dRMS and binding energy are plotted as functions of time for each simulation between the actin and the Arp2/3 complex. Representative conformations are displayed. Simulations performed in cubic boxes of side 300 Å for 1 μs.

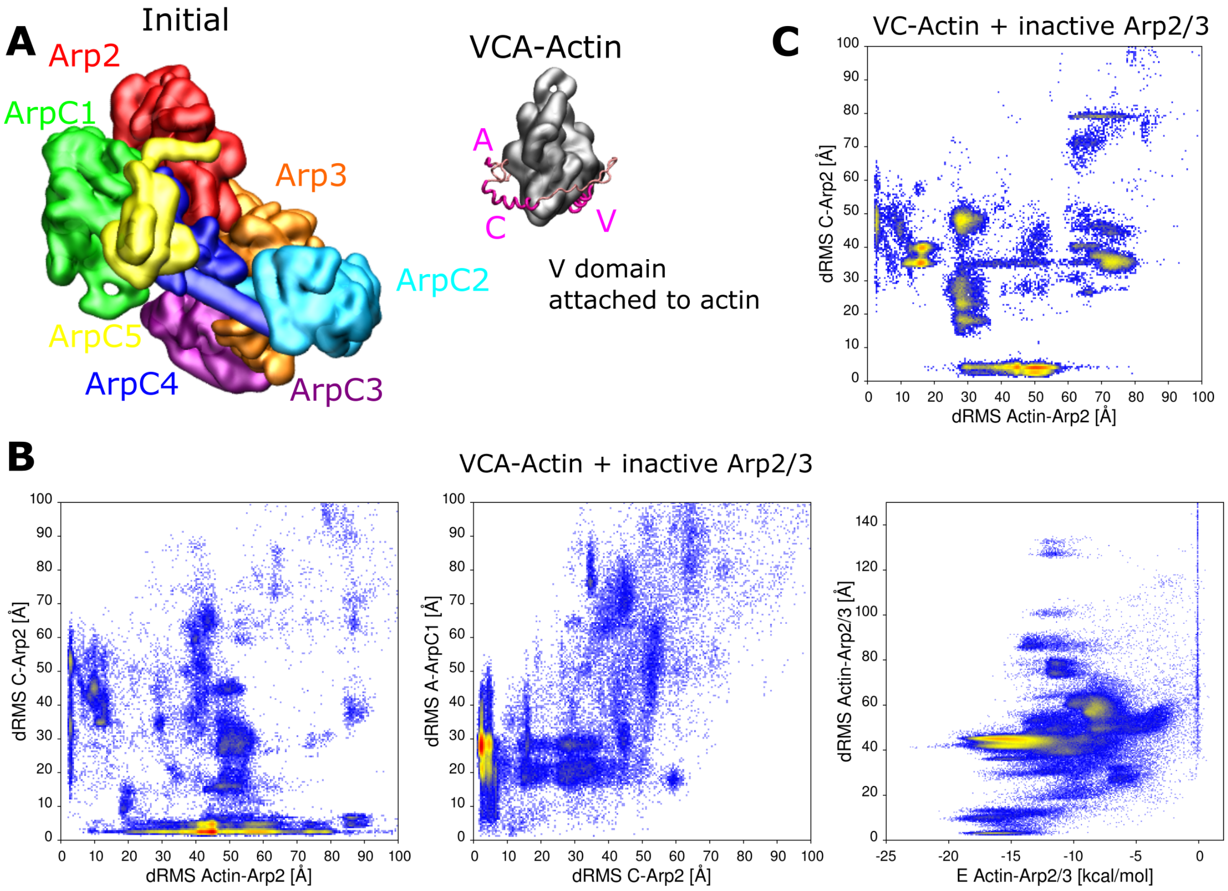

Figure S7. Simulation of VCA-actin and VC-actin binding to inactive Arp2/3 complex. (A) Snapshot of REMD simulation of VCA-actin binding to inactive Arp2/3 complex. The VCA domain is attached to an F-actin subunit with flexible D-loop through the V region, initially separated from the Arp2/3 complex. The C domain is held rigid in the helical configuration of the Arp2-ArpC1 site as in Figure 1B and 3C. **(B)** dRMS and energy plots for VCA-actin and inactive Arp2/3 complex. Hotspots with low dRMS indicate binding modes close to the reference configurations. The dRMS of C and A regions are calculated with respect to the Arp2-ArpC1 site, as in Figure 1A and 3C, and of actin with respect to long-pitch binding to Arp2, as in Figure 3A. Left: 2D distribution of dRMS values for actin and C domain relative to Arp2. Middle: 2D distribution of dRMS values for C and A domains relative to their Arp2-ArpC1 binding sites. Right: 2D distribution of dRMS vs interaction energy between actin and Arp2/3 complex. Data binned over 0.5×0.5 Å^2 or 0.1 kCal/mol $\times$ 0.5 Å. **(C)** Same as first plot of panel B, but for VC-actin instead of VCA-actin. VC domain is the same as in Figures 2, 3A and 3B (R431-I481). Both VCA-actin and VC-actin simulations performed in cubic boxes of side 300 Å for $\sim$ 1.8 μs and $\sim$ 1.4 μs respectively.

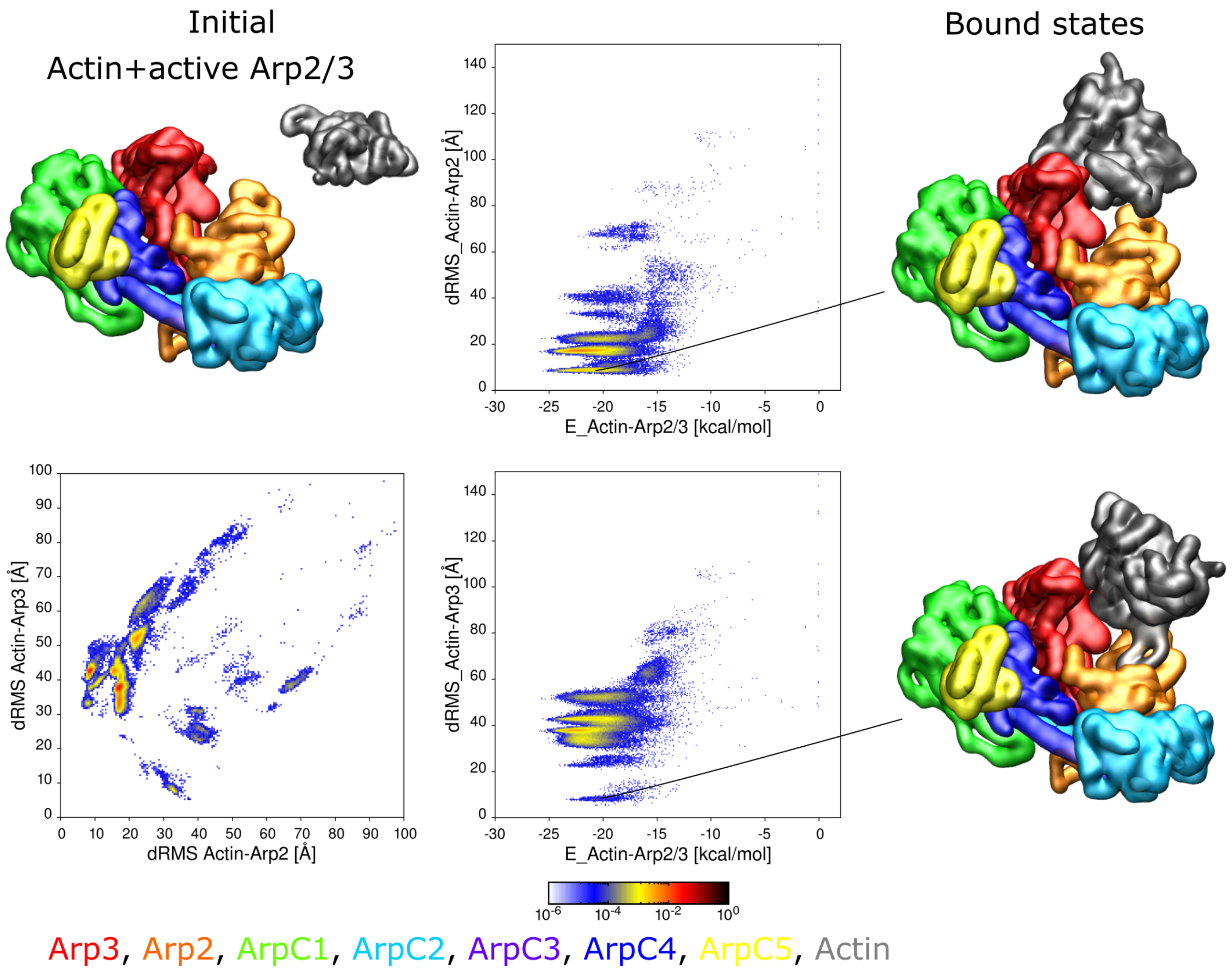

Figure S8. Delivery of actin to the active Arp2/3 complex without VC domain. REMD simulations of actin and active Arp2/3 complex starting from separate state (snapshot). The 2D- distributions of dRMS and binding energies are calculated and plotted in bins of 0.1 kCal/mol $\times$ 0.5 Å or 0.5 $\times$ 0.5 Å^2. The bound conformations are randomly selected the from the hotspot with the lowest dRMS values. Simulation performed in a cubic box of side 300 Å for $\sim$ 900 ns.

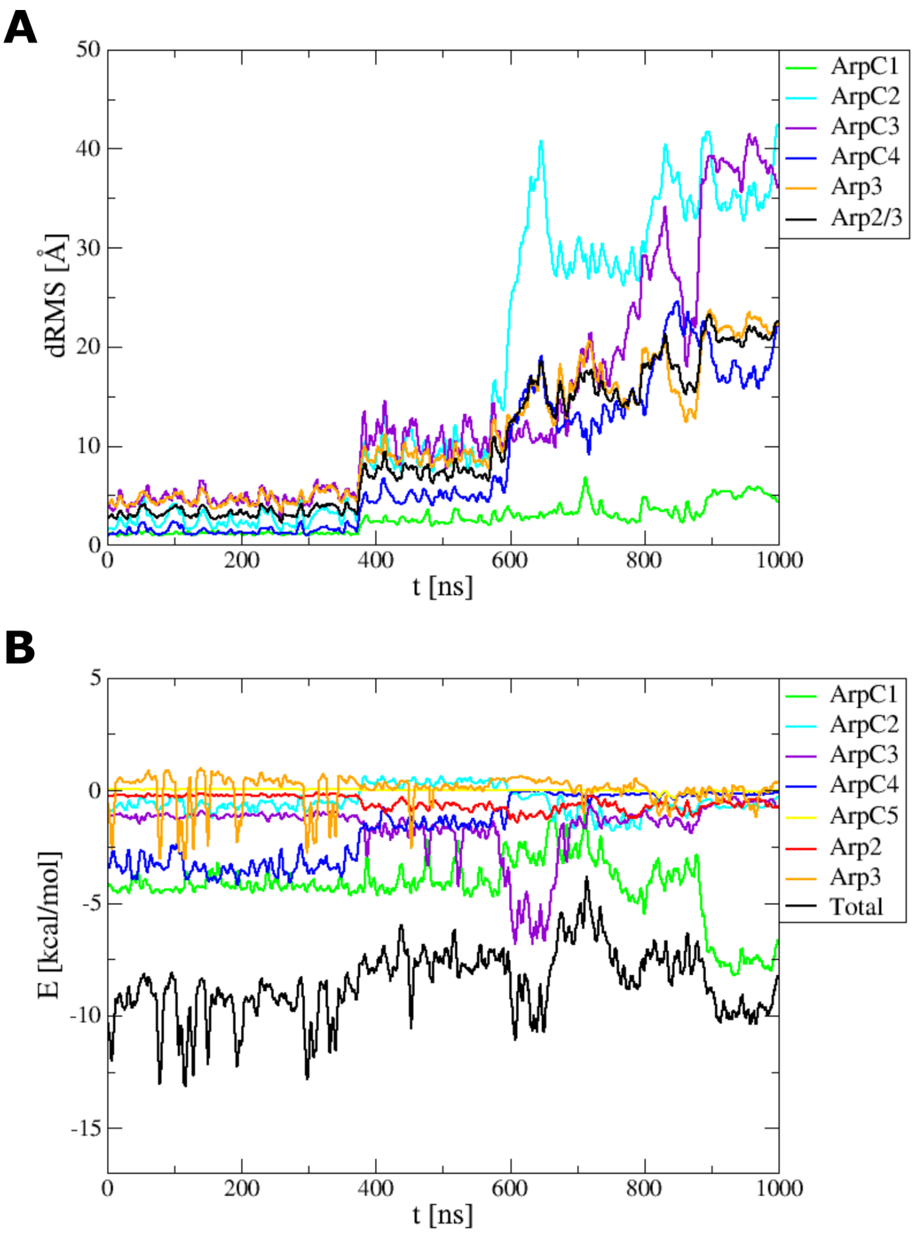

Figure S9. Simulation of active Arp2/3 complex bound to a mother actin filament without ArpC2 C-terminal tail. Serial simulation as of Arp2/3 complex interacting with a mother actin filament as in Figure 5C,D, but with the Arp2 C-terminal tail left missing. **(A)** dRMS of Arp2/3 complex subunits in contact with mother actin filament versus time. **(B)** Interaction energies of Arp2/3 complex subunits and of whole complex with mother filament versus time. Simulation shows quick movement of Arp2/3 complex away from its initial position as compared to the case with the ArpC2 tail included (Figure 5).

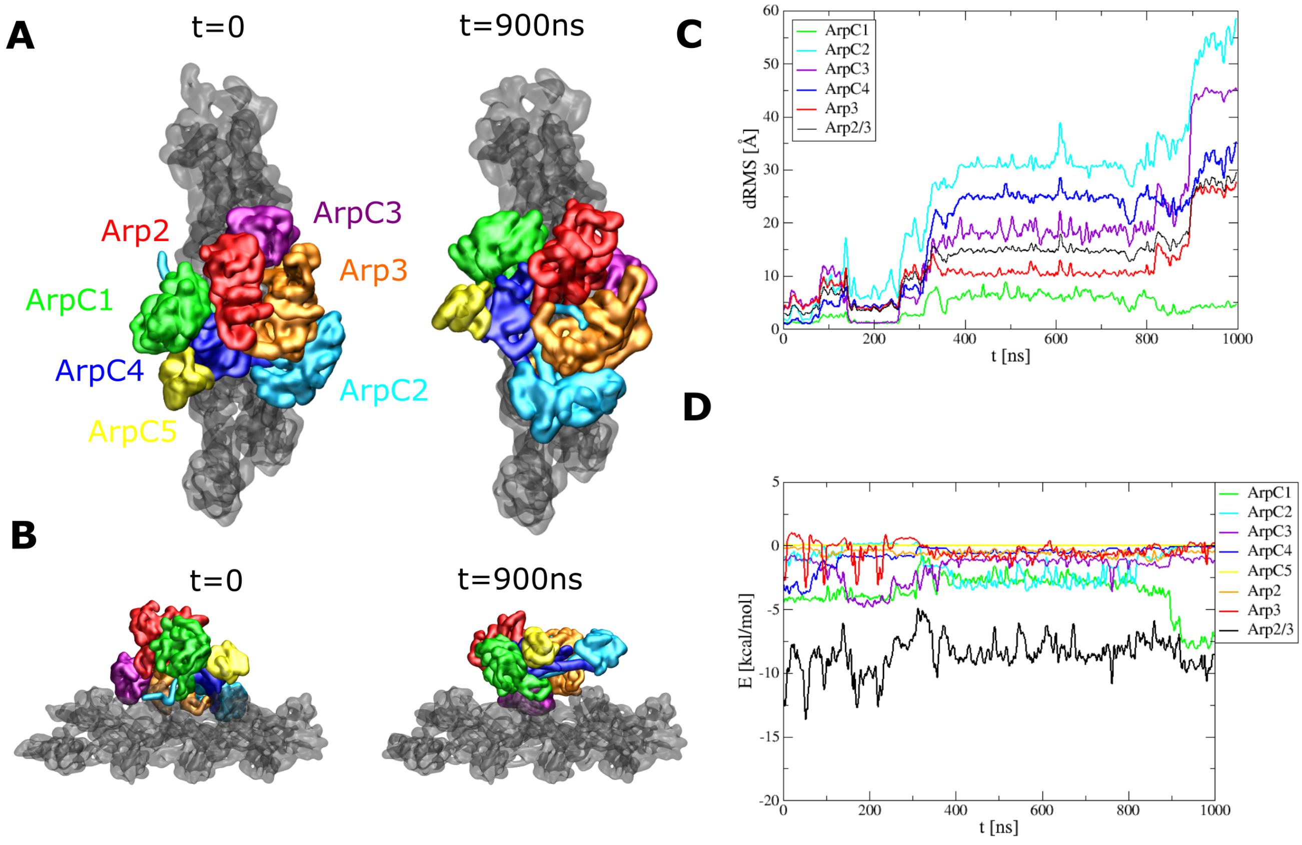

Figure S10. Arp2/3 complex bound to actin filament with ArpC2 tail built between ArpC1 and ArpC3. (A,B) Serial simulation starting from Arp2/3 complex bound to the mother filament as in Figure 5. The unstable binding conformation at 900 ns is displayed in top and side views. **(C,D)** dRMS and binding energy for each subunit and the entire Arp2/3 complex is plotted as a function of time.

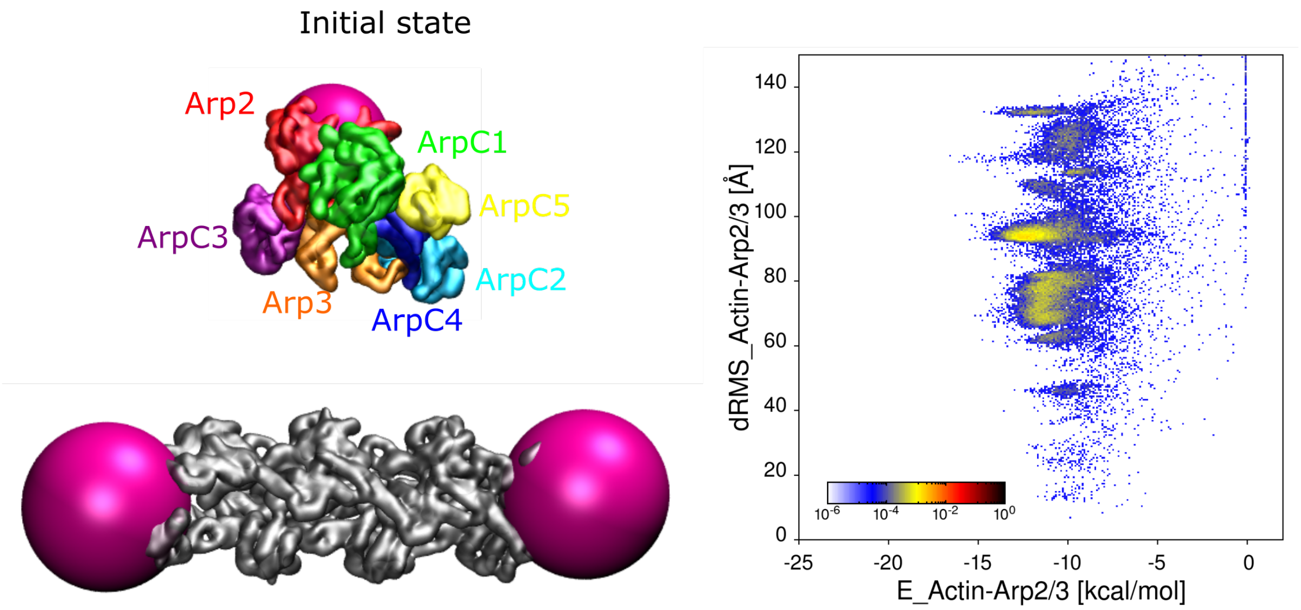

Figure S11. REMD simulation of active Arp2/3 complex and mother actin filament starting from separate state. The Arp2/3 complex and mother actin filament were taken from PDB 7AQK and treated as separate rigid bodies except for the D-loops of the mother actin filament. Unlike Figure 5, Arp2 and Arp3 were set fully rigid. The LAMMPS mass variable of Arp2/3 complex was reduced to allow faster diffusion of the complex. To prevent binding of Arp2/3 complex to mother filament ends, or binding of the barbed ends of Arp2/Arp3 with mother actin filament, repulsive beads (magenta spheres) were added to the ends of the filament and in between Arp2 and Arp3. These beads interact with all other atoms in the simulation through a repulsive only LJ potential of ϵ 10.0 kCal/mol, and σ and cutoff both 42.5 Å. The Arp2/3 complex interface with Arp2/Arp3 barbed end, which binds the daughter actin filament, was also capped with a repulsive bead interacting with other atoms through a similar repulsive LJ potential of the same ϵ and cutoff of 34 Å. The interaction ranges were selected to properly cap the corresponding interface. In simulations without the addition of repulsive beads, such interactions were occurring with large probability. The size of the spheres reflect the range of the repulsive interaction. Right: 2D distribution of dRMS vs binding energy, evaluated with respect to the 7AQK reference. The Arp2/3 complex associated with the actin filament in several orientations indicated by several hotspots. We did not observe a low dRMS peak, presumably because the interaction energy in the 7AQK reference is weaker without the ArpC2 tail (compare Figure 5D to Figure S7B). Simulation performed in a cubic box of side 400 Å for 400 ns. The simulation was repeated four more times with different starting random seeds, giving similar results.
